# Cytochalasin B Modulates Nanomechanical Patterning and Fate in Human Adipose-Derived Stem Cells

**DOI:** 10.3390/cells11101629

**Published:** 2022-05-12

**Authors:** Eva Bianconi, Riccardo Tassinari, Andrea Alessandrini, Gregorio Ragazzini, Claudia Cavallini, Provvidenza Maria Abruzzo, Giovannamaria Petrocelli, Luca Pampanella, Raffaella Casadei, Margherita Maioli, Silvia Canaider, Federica Facchin, Carlo Ventura

**Affiliations:** 1 Laboratory of Cardiovascular Biology, IRCCS Ospedale Policlinico San Martino, Viale Rosanna Benzi 10, 16132 Genova, Italy; eva.bianconi2@gmail.com; 2 National Laboratory of Molecular Biology and Stem Cell Bioengineering of the National Institute of Biostructures and Biosystems (NIBB)—Eldor Lab, Innovation Accelerator, CNR, Via Piero Gobetti 101, 40129 Bologna, Italy; riccardo.tassinari.rt@gmail.com (R.T.); clo.cavallini@gmail.com (C.C.); carlo.ventura@unibo.it (C.V.); 3 Department of Physics, Informatics and Mathematics, University of Modena and Reggio Emilia, Via Campi 213/A, 41125 Modena, Italy; andrea.alessandrini@unimore.it (A.A.); gregorio.ragazzini@unimore.it (G.R.); 4 CNR-Nanoscience Institute-S3, Via Campi 213/A, 41125 Modena, Italy; 5 Department of Experimental, Diagnostic and Specialty Medicine (DIMES), University of Bologna, Via Massarenti 9, 40138 Bologna, Italy; provvidenza.abruzzo2@unibo.it (P.M.A.); giovannam.petrocell2@unibo.it (G.P.); luca.pampanella@studio.unibo.it (L.P.); 6 Department for Life Quality Studies (QuVi), University of Bologna, Corso D’Augusto 237, 47921 Rimini, Italy; r.casadei@unibo.it; 7 Department of Biomedical Sciences, University of Sassari, Viale San Pietro 43/B, 07100 Sassari, Italy; mmaioli@uniss.it

**Keywords:** cytoskeleton, human adipose-derived stem cells, cytochalasin B, actin microfilaments, atomic force microscopy, nanomechanics, adipogenic commitment

## Abstract

Cytoskeletal proteins provide architectural and signaling cues within cells. They are able to reorganize themselves in response to mechanical forces, converting the stimuli received into specific cellular responses. Thus, the cytoskeleton influences cell shape, proliferation, and even differentiation. In particular, the cytoskeleton affects the fate of mesenchymal stem cells (MSCs), which are highly attractive candidates for cell therapy approaches due to their capacity for self-renewal and multi-lineage differentiation. Cytochalasin B (CB), a cyto-permeable mycotoxin, is able to inhibit the formation of actin microfilaments, resulting in direct effects on cell biological properties. Here, we investigated for the first time the effects of different concentrations of CB (0.1–10 μM) on human adipose-derived stem cells (hASCs) both after 24 h (h) of CB treatment and 24 h after CB wash-out. CB influenced the metabolism, proliferation, and morphology of hASCs in a dose-dependent manner, in association with progressive disorganization of actin microfilaments. Furthermore, the removal of CB highlighted the ability of cells to restore their cytoskeletal organization. Finally, atomic force microscopy (AFM) revealed that cytoskeletal changes induced by CB modulated the viscoelastic properties of hASCs, influencing their stiffness and viscosity, thereby affecting adipogenic fate.

## 1. Introduction

Recent evidence highlights that cytoskeletal dynamics play a pivotal role in mechano-sensing, mechano-transduction [1], and in organization of bioelectrical properties of cells [2,3,4]. The cytoskeleton acts as a bioelectronic circuit with profound influences on cell shape, proliferation, and even differentiation [5,6,7].

In particular, the cytoskeleton affects mesenchymal stem cell (MSC) fate. MSC differentiation is accompanied by changes in mechanical properties according to their lineage. These changes are already evident during the first hours (h) of stem cell differentiation, and are linked to variations in gene expression profiles [8]. A somewhat disorganized and less stiff actin cytoskeleton is a necessary feature to differentiate MSCs into adipocytes [9,10]. In fact, MSCs are more prone to adipogenic commitment when the cells are rounded and actin polymerization or cell spreading is inhibited. Moreover, both adipogenesis and chondrogenesis are encouraged by preventing focal adhesion attachment [11], and chondrogenesis seems to be favored by decreased actin cytoskeletal organization, an event associated with a reduction in Ras homolog family member A activity [12]. On the contrary, osteogenesis requires a large number of focal adhesions and a stiff, spread actin cytoskeleton [13]. As a result, the speed of osteogenic differentiation is also affected by the chemical depolymerization of microtubules [8].

Different factors can interfere with cytoskeletal organization: they either disrupt (cytochalasins or lantruculin and vincristine or nocodazole) or rigidify (jasplakinolide and paclitaxel) actin microfilaments and microtubules, respectively [1,14], by altering their dynamic properties. In particular, cytochalasins (from the Greek cytos-cell and chalasis-relaxation) are a family of 60 metabolites, isolated for the first time in the late 1960s. They are produced by different species of fungi [15,16,17] and are able to act on the cytoskeleton, producing different effects on the basis of the molecular mechanism exploited. Among all the cytochalasins, only cytochalasins D (CD) and B (CB) interfere with actin dynamics and have reversible action. In fact, when they are removed, their effects on the cytoskeleton are lost [18]. In addition, the same dose of these cytochalasins may reduce F-actin content in one cell type but not in others [19], or specifically affect different F-actin domains within a given cell type [20,21]. In particular, the effects of CD may vary in a dose- and cell-type-dependent manner. In several cell types, an inverse relationship has been observed between CD concentration employed and the cellular content of F-actin. The presence of actin depolymerization was found with low doses of toxin, and conversely, an absence of depolymerization or even increased polymerization with higher doses [22,23].

While CD and its effects on different cell processes and cell models have already been extensively studied, the role of CB deserves further investigation. CB is a cyto-permeable mycotoxin, and it is isolated from an ascomycete fungus belonging to the *Phoma* genus [24,25], whose rhabdomyolytic role has been known since the 80s. It has been shown that sub-stoichiometric concentrations of this toxin are able to inhibit the formation of actin microfilaments through a direct effect on the polymerization of the cytoskeletal protein [26]. This toxin, by binding the fast-growing end of F-actin and interacting with capping proteins (i.e., F-actin-capping protein subunit alpha-1), influences the nucleation-elongation of actin microfilaments [27]. CB, acting on the cytoskeleton, can interfere with cell division, blocking the formation of the actin contractile ring [25] and stopping the cell cycle in the G2 to M transition phase. CB makes cells unable to properly form their mitotic spindles [28] and induces cells to enter the G0 resting state until sufficient actin levels are restored [29]. In fact, since the effect of CB is reversible, its removal induces the reactivation of the cell cycle [29] and of cytokinesis [25]. Compounding the pleiotropic consequences of CB-mediated inhibition of actin polymerization, this toxin has also been found to: (i) alter cell migration, through actions involving, besides the direct effect on actin, cytosolic calcium handling and redistribution patterning of other cytoskeletal elements [30]; (ii) inhibit developmental commitments, such as the acquirement of a dendritic fate in B lymphocyte precursors exposed to 4 beta-phorbol 12-myristate 13-acetate [31].

Several studies have investigated the role of cytochalasins in human MSCs (hMSCs). These toxins, by interfering with the organization of the actin cytoskeleton, significantly modulate the differentiation potential of hMSCs [11]. In particular, CD was found to promote osteogenesis in bone marrow-derived stem cells (BM-MSCs) [32,33], whereas it either inhibited or enhanced this effect in human adipose-derived stem cells (hASCs) when used alone or in association with other agents [34,35,36,37]. At the same time, several studies demonstrated that CD was able to stimulate adipogenesis in different hMSC models [9,38,39]. On the other hand, few results are known regarding the role of CB in MSC commitment. To this end, the capability of CB to act as a glucose uptake inhibitor in human periodontal ligament-derived MSCs that had been primed for neurogenic differentiation led to a decrease in neurosphere formation efficiency [40].

Therefore, aim of this study was to explore for the first time the effects of CB on adipogenic ability and on a number of other nanomechanical and biological properties of hASCs. These cells exhibit multi-lineage differentiation and trophic signaling that make them highly attractive candidates for cell therapy approaches [41]. In fact, hASCs are commonly harvested in less invasive contexts than others, and have robust multipotency and rapid proliferation [42]. Moreover, hASCs are not burdened by ethical problems and are supposed to be safer than embryonic stem cells or induced pluripotent stem cells in terms of tumorigenesis [43].

We first assessed the effects of CB on hASC metabolism, viability, proliferation and morphology, investigating the dose-dependency of cellular responses, and the capability of hASCs to retrieve their morphology and proliferative activity upon toxin withdrawal from the culture medium. Within this context, we aimed at dissecting CB’s mechanisms of action on the expression and subcellular redistribution profiles of F-actin and vinculin, two selected components of the focal adhesion complexes (FACs). Afterwards, we analyzed the effects of the chosen CB concentrations on the ability of hASCs to undergo an adipogenic commitment, by analyzing the changes in the expression of tissue-restricted players, the presence of a lipid droplet-associated protein (perilipin), and the formation of lipidic vacuoles. Based upon the inverse relationship between hMSC stiffness and adipogenic commitment [9], we designed specific experiments with atomic force microscopy (AFM) to elucidate whether CB affects hASCs’ rheological properties at the nanoscale, and whether, if so, this mycotoxin may be exploited as a tool for finely tuning hASC elasticity and viscosity, two fundamental traits in hASC adipogenesis.

## 2. Materials and Methods

### 2.1. hASCs: Harvesting and Culture

Tissue samples were obtained after informed consent from 3 healthy female subjects that experienced esthetical liposuction. The study was approved by the Institutional Ethics Committee Sant’Orsola-Malpighi University Hospital of Bologna, project identification code EM468-2019, reference 6/2016/U/Tess/AOUBo, 22/05/2019. Briefly, aliquots of lipoaspirates were digested with Collagenase II 0.075% (Sigma-Aldrich Co., St. Louis, MO, USA) at 37 °C for 30 min with gentle agitation and centrifuged to eliminate oil. Pellets were treated with a hemolytic solution (VersaLyse; Beckman Coulter, Brea, CA, USA) and then centrifuged. The stromal-vascular fractions obtained after centrifugation were plated in 175 cm^2^ flasks (Corning Incorporated, Corning, NY, USA) at the density of 25,000 cells/cm^2^. Cells were cultured in Dulbecco’s modified eagle’s medium—1 g/L of glucose (L-DMEM, Corning Incorporated, Corning, NY, USA) supplemented with 10% fetal bovine serum (FBS; Gibco, Waltham, MA, USA) and antibiotics (1% Penicillin-Streptomycin Solution; Thermo-Fisher Scientific, Waltham, MA, USA) and were maintained in standard culture conditions at 37 °C with 5% carbon dioxide (CO_2_) in a humidified atmosphere. Medium was replaced after 4 days from the seeding to allow hASCs to adhere to the flask, and then twice a week until confluence. When reaching 80% confluence, hASCs were passaged by treatment with trypsin–EDTA (Sigma-Aldrich Co., St. Louis, MO, USA). In each experiment, cells were seeded at a density of 4000 cells/cm^2^ (except for adipogenic commitment and osteogenic/chondrogenic commitment analyses where the seeding density was 9000 or 5000 cells/cm^2^, respectively) with adequate plastic support (Corning Incorporated, Corning, NY, USA) and incubated in standard condition for 24 h before the treatment. Experiments were performed using hASCs on the 3rd–6th culture passage from three human samples (*n* = 3).

### 2.2. hASC Characterization

The hASCs’ phenotype and their trilineage differentiation potential were assessed.

To evaluate the expression of surface markers, cells were harvested with trypsin-EDTA, washed in phosphate buffered saline (PBS, Sigma-Aldrich Co., St. Louis, MO, USA), and fixed with 4% formaldehyde (Sigma-Aldrich Co., St. Louis, MO, USA) for 10 min. After then, cells were incubated with fluorescein isothiocyanate (FITC) or phycoerythrin (PE)-conjugated monoclonal antibodies against CD90, CD105, CD73, CD45, and CD34 (eBioscienceTM, Thermo Fisher Scientific, San Diego, CA, USA) for 30 min at 4 °C in darkness. Cells were washed with PBS to remove unbound antibodies. For each condition, a total of 10,000 viable cells (events) were acquired using a Partec flow cytometer instrument (Sysmex Partec GmBH, Görlitz, Germany); data were analyzed by using the FlowJo software (Tree Star, Ashland, OR, USA).

The adipogenic potential was evaluated as described in Section 2.11.

In order to test the osteogenic potential, when hASCs reached 70–80% confluence, osteogenesis was induced by a “StemPro Osteogenesis Differentiation Kit” (Thermo Fisher Scientific, Waltham, MA, USA), following the manufacturer’s recommendations. The osteogenic medium was changed every 3–4 days for 21 days. At the end of the differentiation protocol, cells were washed in PBS, fixed in 4% formaldehyde for 15 min, and stained with Alizarin stain (Sigma Aldrich, St. Louis, MO, USA) to detect Ca^2+^ deposits.

To assess the chondrogenic potential, the cells were cultured in standard conditions until they reached 90% confluence. Then, the standard medium was aspirated and replaced with complete MesenCult-ACF Chondrogenic Differentiation Medium (StemCell Technologies, Vancouver, CA-BC, Canada). During 21 days of differentiation, the chondrogenic medium was changed every 3 days. The formation of cartilage proteoglycans was assessed by Alcian Blue staining (Sigma-Aldrich, St. Louis, MO, USA).

### 2.3. Cytochalasin B treatments

Cytochalasin B (CB) was purchased from Sigma-Aldrich (Cytochalasin B from *Drechslera dematioidea*, catalog number C6762, Sigma-Aldrich Co., St. Louis, MO, USA) and dissolved in dimethyl sulfoxide (DMSO, Sigma-Aldrich Co., St. Louis, MO, USA) at 10 mg/mL. The stock solution was conserved at −20 °C until the day of the experiment, when it was appropriately diluted in L-DMEM. CB was used at different concentrations (0.1, 1, or 10 μM). An additional concentration of CB 5 μM was investigated with atomic force microscopy (AFM) and adipogenic analyses. DMSO (the CB vehicle) treatment was included at the final concentration of 0.05%. In each experiment, untreated cells were used as control (CTR).

### 2.4. Resazurin-Based Assay

In order to assess hASC metabolism, cells were seeded in a 96-well plate (Corning Incorporated, Corning, NY, USA) and the “In vitro toxicology assay kit, Resazurin based” (TOX8, catalog number R6892, Sigma-Aldrich Co., St. Louis, MO, USA) was used [44]. Resazurin is reduced by metabolically active cells to the fluorescent molecule resorufin. Resazurin was added concomitant with the treatments, and resorufin fluorescence was investigated 4, 24, 48 and 72 h after the beginning of CB administration. Each condition was assayed in quadruplicate, and fluorescence was recorded with the Wallac 1420 Victor2 Multilabel Counter (Perkin Elmer, Waltham, MA, USA) at 590 nm using an excitation wavelength of 560 nm. Negative controls without cells (i.e., complete medium and Resazurin with or without CB or DMSO) and fully reduced Resazurin were included. hASC metabolism was expressed as percentage of resazurin reduction according to this formula: (FI 590 of test agent-FI 590 of negative control)/(FI 590 of 100% reduced resazurin-FI 590 negative control) × 100, where FI is fluorescence intensity.

### 2.5. Cell Count

hASCs, seeded in a 48-well plate (Corning Incorporated, Corning, NY, USA), were exposed for 24, 72 h (3 days), 6 or 14 days to CB or DMSO. Moreover, hASC recovery was assessed after 24 or 48 h from the end of 24 h CB treatment. At each time point, cells were detached (2 wells/condition for each experimental point) by trypsin–EDTA and counted, as previously described [45]. Briefly, cells were resuspended in a medium with 50% erythrosine B (Sigma-Aldrich Co., St. Louis, MO, USA) and 0.2% red dye in PBS. Not stained viable cells and red stained dead cells were manually counted, at least twice for each condition, using the Neubauer hemocytometer (BRAND GmbH, Wertheim, Germany) and a light microscope (Nikon Eclipse TS100, Nikon Instruments, Melville, NY, USA). The total numbers of viable and dead cells were calculated according to the manufacturer’s instructions; for each sample, cell viability was obtained calculating the percentage of living cells compared to the total number of cells. Moreover, cell growth rate (gr) was calculated using the following formula: gr = (ln (N(t)/N(0))/t, where N(t) = the number of cells counted after 24 h or 48 h recovery upon CB removal; N(0) = the number of cells counted after 24 h of CB treatment; t = time passed (24 or 48 h).

### 2.6. RNA Extraction, RT-PCR and Real Time-PCR

hASCs, seeded in 25 cm^2^ flasks (Corning Incorporated, Corning, NY, USA), were exposed to CB at the specific concentration of 1 μM for 24 h. At the end of the treatment and after 24 h of recovery after the chemical’s removal, total RNA was extracted from treated and untreated (CTR) hASCs using the RNeasy Mini Kit (QIAGEN, Valencia, CA, USA) and digested with RNase-free Deoxyribonuclease I (RNase-free DNase set, QIAGEN, Valencia, CA, USA) following the manufacturer′s instructions. Then, 800 ng of RNA template was reverse-transcribed in cDNA using the iScript™ RT Supermix (Bio-Rad Laboratories, Inc., Hercules, CA, USA) according to the manufacturer’s instructions. To verify the retrotranscription reaction, *glyceraldehyde 3-phosphate dehydrogenase* (*GAPDH*) gene amplification was performed as described in Facchin and colleagues (2011) [46], except for 40 cycles instead of 45; *GAPDH* amplicon detection was performed by gel agarose electrophoresis, as described by Beraudi and collaborators (2016) [47].

For each experimental condition, 25 ng of cDNA was amplified using the SsoAdvanced Universal SYBR Green Supermix (Bio-Rad Laboratories, Hercules, CA, USA) in technical triplicates in a Bio-Rad CFX96 real-time thermal cycler (Bio-Rad Laboratories, Hercules, CA, USA), as previously described [46].

The expression levels of *cyclin dependent kinase inhibitor 1A (CDKN1A*, *alias p21*), *cyclin dependent kinase inhibitor 2A (CDKN2A*, *alias p16^INK4α^*); *MKI67*, encoding for the marker of proliferation Ki-67; and *CCND1*, encoding for the cell cycle controller Cyclin D1, were determined by CFX Manager Software version 3.1 (Bio-Rad Laboratories, Hercules, CA, USA) using the “delta-delta CT method” [48,49]. Data were normalized using four reference genes: *GAPDH*, *TATA box binding protein* (*TBP*), *ribosomal protein L13* (*Rpl13*), and *hypoxanthine phosphoribosyl transferase 1* (*HPRT1*). Primer sequences for *CDKN1A*, *CDKN2A*, *HPRT1*, and *TBP* were designed by Bio-Rad (20X, ID numbers: qHsaCED0056722, qHsaCID0014498, qHsaCIP0030549, and qHsaCIP0036255, respectively, Bio-Rad Laboratories, Hercules, CA, USA) and were used in accordance with the manufacturer’s instructions. The primer sequences for *MKI67* (forward primer sequence—tcagactccatgtgcctgag; reverse primer sequence—ttgtcctcagccttctttgg), *CCND1* (forward primer sequence—cagatcatccgcaaacacgc; reverse primer sequence—aagttgttggggctcctcag), *GAPDH* (forward primer sequence—cctgacctgccgtctagaaa; reverse primer sequence—tgctgtagccaaattcgttg), and *Rpl13* (forward primer sequence—tgaaggagtaccgctccaaac; reverse primer sequence—ggagactagcgaaggctttga) were designed using the PrimerBlast and the Amplify tools and were purchased from Sigma-Aldrich (Sigma-Aldrich Co., St. Louis, MO, USA). For each gene, the normalized expression value of untreated cells (CTR) was set to 1, and all other gene expression values are reported with respect to CTR. Data are expressed as fold change ± standard error of the mean (SEM).

### 2.7. Morphological Analysis and MTT Assay

In order to analyze hASC morphology, cells were seeded in 48-well plates and exposed for 24 h to CB or DMSO. Every experimental condition was assayed in technical duplicate. At the end of the 24 h treatment and an additional 24 h after the chemical’s removal, a morphological analysis was performed, and cells were observed under a light microscope (Nikon Eclipse TS100, Nikon Instruments, Melville, NY, USA) and a digital sight camera DS-Fi1 in bright field (Nikon Instruments, Melville, NY, USA). In parallel to the morphological analysis, the 3-(4,5-dimethylthiazol-2-yl)-2,5-diphenyltetrazolium bromide (MTT) colorimetric assay was performed. MTT is a soluble tetrazolium dye that is reduced by living cells in a not soluble form, the formazan. The magnitude of the MTT reduction in formazan is commonly used as a cell metabolic marker. To perform this assay, at the end of 24 h CB treatment and 24 h after the chemical’s removal, a solution of MTT 5 mg/mL in PBS was added to every sample at the ratio of 1:10 for the final volume (i.e., medium with or without treatment). After 3 h incubation, formazan was solubilized with sodium dodecyl sulfate (SDS, Sigma-Aldrich Co., St. Louis, MO, USA), 10%, in HCl 0.01 M (250 μL/well). The solubilized salt of every experimental condition was transferred in duplicate in a 96-well plate. Absorbance at 570 nm was measured using the plate reader Multiskan Go (Thermo-Fisher Scientific, Waltham, MA, USA).

### 2.8. Live Cell Imaging

The effect of CB treatment and of its removal on cell morphology was also evaluated by time-lapse live cell imaging. This method was based on the use of an on-stage incubator coupled to an inverted optical microscope (Olympus IX 70, Tokyo, Japan) [50]. The incubator was placed near the microscope condenser, and the whole apparatus was protected from the light. The internal conditions of the incubator were: temperature at 37 °C, 90–95% humidity, and 5% CO_2_. hASCs, seeded in a 6-well plate (Corning Incorporated, Corning, NY, USA), were placed inside the on-stage incubator and were treated with the medium containing the different concentrations of CB for 7 h. In addition, to study the effects of the chemical’s removal, the culture medium was replaced with fresh complete medium after 2/3 or 1/3 of the incubation time for the cells treated with CB 1 μM or CB 10 μM, respectively. Throughout the period of analysis, a series of images were acquired automatically by a system based on a home-developed autofocus algorithm and software based on the Python programming language. Images were then used to obtain a movie of 7 s for each experimental condition.

### 2.9. Immunofluorescence of Cytoskeletal Markers

hASCs were seeded in a 24-well plate (Corning Incorporated, Corning, NY, USA) and treated for 24 h or 3 days with CB or DMSO. Experiments for identification of F-actin and vinculin immunofluorescence were performed at the end of the 24 h or 3 days-treatment and 24 h after CB wash-out. Each experimental condition was performed in technical duplicate. At the defined experimental times, cells were fixed with 4% formaldehyde (Sigma-Aldrich Co., St. Louis, MO, USA) for 15 min, washed with PBS-Tween 0.25% and permeabilized with Triton X-100 0.25% and sodium citrate 10 mM (Sigma-Aldrich Co., St. Louis, MO, USA) in PBS for 15 min at room temperature (RT). Samples were blocked for 1 h with a solution containing 4% bovine serum albumin (BSA; Sigma-Aldrich Co., St. Louis, MO, USA) and 0.3% Triton X-100 in PBS. In order to visualize focal adhesions, firstly, cells were incubated with the primary antibody (AB), anti-vinculin (MAB3574; Chemicon-Sigma-Aldrich Co., St. Louis, MO, USA), for 3 h at RT and then with the appropriate fluorescence-conjugated secondary AB (anti-mouse conjugated with tetramethylrhodamine-5-(and 6)-isothiocyanate—Sigma-Aldrich Co., St. Louis, MO, USA) for 1 h at RT. ABs were diluted in a solution of 2% BSA and 0.15% Triton X-100 in PBS (dilution 1:200). Incubation with NucBlue^®^ Fixed Cell ReadyProbes^®^ Reagent (DAPI, Molecular Probes™, Life Technologies—Thermo-Fisher Scientific, Waltham, MA, USA) was used to counter-stain nuclei. Moreover, hASCs were stained with Phalloidin—FITC (P5282; Sigma-Aldrich Co., St. Louis, MO, USA) or Alexa Fluor™ 647–labeled (Life Technologies—Thermo-Fisher Scientific, Waltham, MA, USA)—which is useful for visualizing F-actin. Fixed cell samples were mounted with antifade AF-400 (Immunological Sciences, Rome, Italy). The detection and acquisition of images were performed using a Nikon Inverted Microscope Eclipse Ti2-E (Nikon Instruments, Melville, NY, USA) and a Digital Sight camera DS-Qi2 (Nikon Instruments, Melville, NY, USA) with the imaging software NIS-Elements.

### 2.10. Atomic Force Microscopy

To perform AFM analysis, hASCs seeded in a 35 mm Petri dish (Corning Incorporated, Corning, NY, USA) were analyzed with the BioScope I microscope equipped with a Nanoscope IIIA controller (Veeco Metrology, Plainview, NY, USA). The measurement of mechanical properties, such as the elasticity and the viscosity, was conducted in contact mode in an aqueous environment, by using a silicon nitride cantilever equipped with a triangular tip and with a nominal spring constant of 0.06 N/m. The cantilever spring constant was calibrated using the thermal noise method [51].

The first analysis was performed for cells treated with DMSO (control sample) or CB, 1 or 5 μM, using the Hertz–Sneddon model for the tip/sample contact mechanics described by the following equation [52]:

(1) $$F=\frac{1.4906\tan\left(\theta \right)}{2\left(1-\nu^{2}\right)}E_{Hertz}\delta^{2}$$

where *θ* is the semi-included angle of the pyramidal tip (axis to face), *ν* is the Poisson ratio (for which we assume a value of 0.5 corresponding to incompressible materials), *E_Hertz_* is the corresponding Young’s modulus, and *δ* is the sample indentation. Maps of the Young’s modulus were obtained using the force-volume mode, recording a set of force–displacement curves from many points on a cell. The Hertz–Sneddon model completely neglects the viscous contribution in the analysis of the force curve’s behavior.

A more extensive analysis was realized for cells treated with DMSO or CB (0.1, 1, 5 and 10 μM) considering viscoelastic models to describe the complete approach and retract portions of the force curve (Supplementary Figure S1 for a representative example). To perform this analysis, we exploited Ting’s model using a procedure similar to the one presented in the work by Efremov et al. [52,53]. In Ting’s model, the linear viscoelastic theory is exploited and a relaxation process has to be assumed for the sample. In this model, to account for specific indentation geometries, the elastic modulus of the sample has to be modified by including hereditary integrals (functions that are based on the past history of the deformation process) that describe the relaxation process. Ting’s solution for a rigid pyramidal indenter on a viscoelastic material is exploited according to the formulas:

(2) $$\left\{\begin{array}{c} F\left(t,\delta \left(t\right)\right)=\frac{1.4906\tan\left(\theta \right)}{2\left(1-\nu^{2}\right)}\overset{t}{\underset{0}{\int }}E\left(t-\xi \right)\frac{\partial \delta^{2}}{\partial \xi }d\xi \;for\;0\leq t\leq t_{m}\\ F\left(t,\delta \left(t\right)\right)=\frac{1.4906\tan\left(\theta \right)}{2\left(1-\nu^{2}\right)}\overset{t_{1}\left(t\right)}{\underset{0}{\int }}E\left(t-\xi \right)\frac{\partial \delta^{2}}{\partial \xi }d\xi \;for\;t_{m}<t\leq t_{ind}\\ with:\overset{t}{\underset{t_{1}\left(t\right)}{\int }}E\left(t-\xi \right)\frac{\partial \delta }{\partial \xi }d\xi =0\;for:\;t_{m}<t\leq t_{ind} \end{array}\right.$$

where *F* is the tip/sample force; *t_ind_* is the time of the entire indentation cycle (approach and retract phases); *t_m_* is the inversion time of the indentation cycle; *t*_1_ is a parameter needed to compensate for the sample relaxation during the retraction phase, and it is calculated by the expression reported in the above formula; and *E*(*t*) represents the Young’s modulus relaxation expression. Traditional relaxation processes such as the ones that can be described by the combination of Maxwell and Kelvin–Voigt elements could be used. However, we found that the process of cell indentation is better described by the power-law model. This model exploits just two cell parameters to describe cell rheology, cell elasticity (stiffness) and cell viscosity or fluidity (the power-law exponent), and it is equivalent to the superposition of infinite spring-dashpot couples connected each other in parallel. Accordingly, for *E*(*t*) we adopted the following expression:

(3) $$E\left(t\right)=E_{0}{\left(\frac{t}{t\prime }\right)}^{-\alpha }$$

where *t′* corresponds to the sampling time of the force curve, and *E*_0_ is the instantaneous Young’s modulus of the sample. The adopted relaxation expression is known as the power-law rheology model, and it is the typically exploited model for AFM rheological measurements of cells [53]. Equation (2) is numerically solved and a least-squares error fitting procedure using the experimental values of the force and is exploited to find the best values for the parameters *E*_0_ and α. To find the representative values of Young’s modulus (*E*_0_) and power-law exponent (α), the distribution of all the subcellular Log*E*_0_ and α values were fitted with Gaussian functions, and the values corresponding to the peak were obtained. All the force-curve analysis was performed with in-house software made using Python. The vertical scanning tip speed was 8 μm/s; the total z-scan displacement was between 2.5 μm and 3 μm. The fitting procedure was repeated for all the sample points of the force volume image (32 × 32 pixels^2^), and maps of the *E*_0_ and α values are reported, where α is expression of the cell viscosity. After acquiring two force volume images of the untreated cells to verify that the system was stable, we injected DMSO or CB in the imaging chamber in order to reach the specified drug concentrations. The cells were then continuously imaged up to a total time of about 2 h while keeping the temperature constant at 37 °C.

### 2.11. Adipogenic Commitment: Immunofluorescence and Oil Red O (O.R.O) Staining

hASCs were seeded at 9000 cells/cm^2^ in 24- or 48-well plates, and medium was changed 2 days after the seeding. On day 4, the complete medium was replaced with StemPro^®^ Adipogenesis Differentiation medium (Thermo-Fisher Scientific, Waltham, MA, USA) alone (CTR) or added with DMSO or CB. The adipogenic protocol induced in cells a slowdown of their proliferation associated with the progressive commitment, as previously discussed [54,55]. As negative (not induced) controls, complete medium supplemented with DMSO or CB at the studied concentrations was added to cells. Media were replaced twice a week for the entire induction protocol (14 days), and at each medium change, the adipogenic or standard medium was supplemented with CB. Each described condition was technically duplicated.

The organization of F-actin was investigated 3, 6, and 14 days after the beginning of the adipogenic protocol in the absence or presence of CB at all the investigated concentrations (included 5 μM), following the method described in Section 2.9.

To evaluate the effects of CB on the expression and localization of early adipogenic markers, the adipogenic protocol was stopped at 3, 6, or 14 days. Cells were fixed with 4% formaldehyde for 25 min, and then incubated with the early adipogenic markers, peroxisome proliferator-activated receptor gamma (PPAR-γ), and CCAAT/enhancer-binding protein alpha (C/EBP-α) ABs (1:200; Cell Signaling Technologies, Danver, MA, USA, #2435 and #8178, respectively) following the protocol described above (see Section 2.9). For each marker and for each experimental condition, ten images were acquired, and the cells positive for each marker were counted; then, the percentage of positive cells was calculated and reported as mean ± standard deviation (SD).

To evaluate the effects of CB on final adipogenic commitment, immunofluorescence analysis of perilipin and O.R.O staining were performed. Perilipin was investigated 3 and 6 days after the beginning of the adipogenic protocol. O.R.O staining was performed at the end of the induced commitment (14th day). For perilipin investigation, hASCs were incubated with anti-perilipin AB (#9349, Cell Signaling Technologies, Danver, MA, USA), following the protocol described above (see Section 2.9). Cells positive for perilipin showed green signal around lipidic vacuoles. For the O.R.O protocol, cells were fixed with 4% formaldehyde in PBS for 45 min at RT and stained with a filtered O.R.O solution (Sigma-Aldrich Co., St. Louis, MO, USA), 0.2% *w*/*v*, in 60% 2-propanol (VWR International, Radnor, PA, USA) for 30 min. Cells positive for adipogenesis showed red vacuoles in the cytoplasm. Image acquisition for O.R.O staining was performed under phase contrast illumination with the Leica MC170 HD Imaging System (Leica Microsystems Srl, Buccinasco, Italy). For both perilipin and O.R.O staining, five images were acquired for each well of each experimental condition. Unstained undifferentiated and stained differentiated cells were counted using ImageJ software [56]. The percentage of stained cells was calculated for each experimental condition and data are reported as the mean of the percentage of positive cells ± SD. Finally, in order to compare the adipogenic commitment between not induced samples (negative controls) and induced samples (samples treated with StemPro^®^ Adipogenesis Differentiation medium), the O.R.O staining was extracted by incubation with isopropanol 100% for 10 min with moderate agitation. For each well, the dye was aliquoted and transferred in duplicate to a 96-well plate prior to read absorbance at 495 nm using a spectrophotometer (Victor 2, Perkin Elmer Wallac, Milan, Italy).

### 2.12. Statistical Analysis

Data were tested for normality, following which, appropriate parametric tests (One-Way ANOVA and post-hoc Tukey Test) or nonparametric equivalents (Kruskal–Wallis test with post-hoc Dunn′s test) were used. Results are shown as mean ± SD. A *p* value < 0.05 was considered statistically significant.

## 3. Results

### 3.1. hASC Surface Markers and Trilineage Differentiative Potential

**Figure 1:**
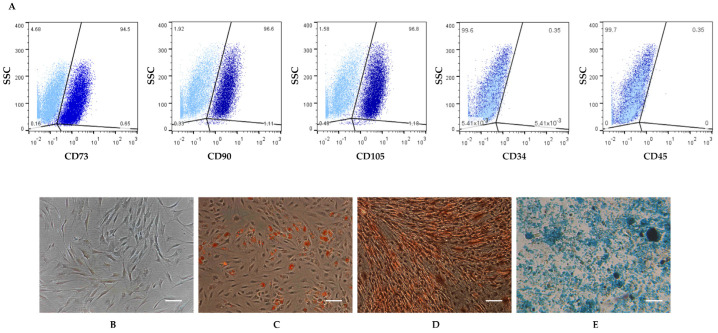
Characterization of human adipose-derived stem cells (hASCs). (**A**) hASC immunophenotypical analysis by flow cytometry. Cells were positive for the expression of the mesenchymal CD73, CD90, and CD105 surface markers, but they did not express the hematopoietic CD34 and CD45 markers. (**B**) hASCs undifferentiated. (**C**–**E**) The differentiation potential of hASCs in: (**C**) adipocytes with intracellular lipid vacuoles visible after Oil Red O (O.R.O) staining; (**D**) osteoblasts with matrix in red after Alizarin Red staining; (**E**) chondrocytes stained with Alcian Blue. Scale bars: 50 μm.

The isolated cells were first characterized for the stem cell markers using flow cytometry. hASCs expressed the major surface markers of MSCs (CD73, CD90, CD105), whereas the hematopoietic related surface markers (CD34 and CD45) were not detected (Figure 1A). Moreover, hASCs showed the typical fibroblast-like shape of MSCs (Figure 1B), and they were able to differentiate toward adipogenic, osteogenic, and chondrogenic lineages when they were cultured with appropriate differentiation media (Figure 1C–E).

### 3.2. Effects of a Prolonged Treatment with Cytochalasin B in hASCs

**Figure 2:**
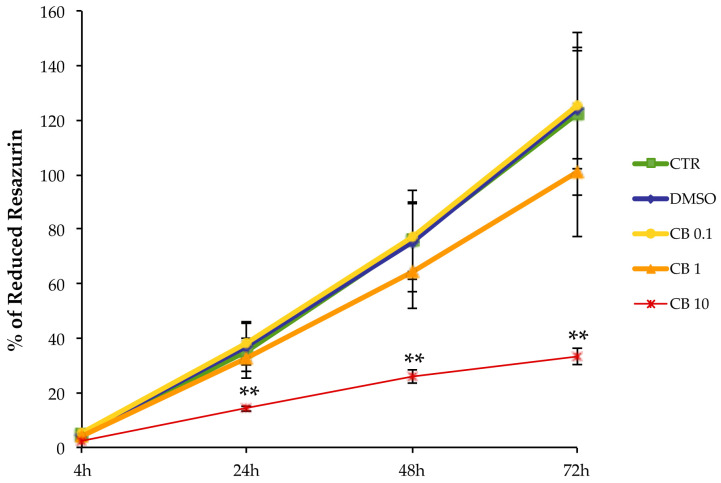
Metabolic activity of human adipose-derived stem cells (hASCs) treated with different concentrations of cytochalasin B (CB). Metabolic activity of hASCs seeded at 4000 cells/cm^2^ was assessed after treatment with 0.1, 1, or 10 μM CB, or 0.05% dimethyl sulfoxide (DMSO) (CB vehicle), or without treatment (CTR). Resazurin reduction was measured at 4, 24, 48 and 72 h from the beginning of the treatment. Data are expressed as mean percentages of reduced resazurin ± standard deviations (SD), *n* = 3; ** *p* < 0.01 vs. CTR.

In order to evaluate the effects of CB on cellular metabolism, hASCs were assessed with the “In vitro toxicology assay kit, Resazurin based” assay in the absence or presence of 0.1, 1, or 10 μM CB or 0.05% DMSO (the CB vehicle). In particular, the continuous presence of CB in the culture medium of hASCs over a time-course ranging from 4 to 72 h, elicited a dose-dependent reduction in resazurin metabolism over each experimental period, compared to the control (CTR) untreated cells. This trend was particularly evident at a concentration of 10 μM CB (*p* < 0.01 starting from 24 h of treatment) (Figure 2). Moreover, cells treated with DMSO showed no statistically significant differences in cell metabolism, compared to the CTR cells (Figure 2).

Based on these results, in the following experiments we decided to mainly investigate the effects of a 24 h treatment with CB on hASCs.

### 3.3. Effects of Cytochalasin B on hASC Viability, Growth Rate, and Cell Cycle Progression

**Figure 3:**
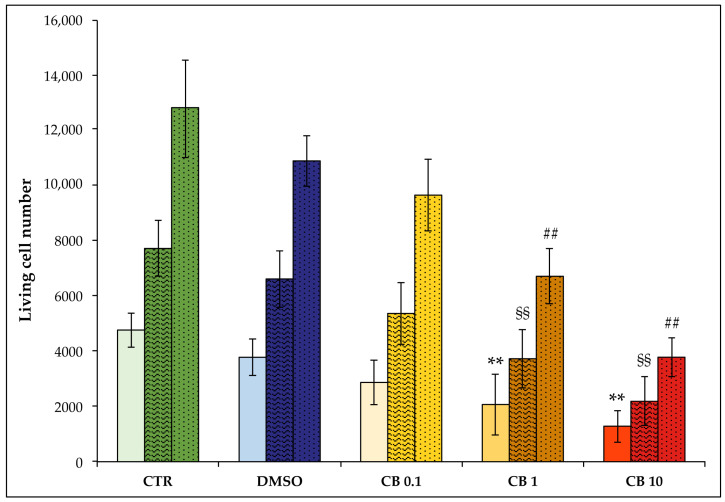
Cell count of living human adipose-derived stem cells (hASCs) after 24 h cytochalasin B (CB) treatment and after a 24 or 48 h recovery time. The number of hASCs seeded at 4000 cells/cm^2^ was assessed after 24 h of treatment with CB at 0.1, 1, and 10 μM, or 0.05% dimethyl sulfoxide (DMSO) (CB vehicle), or without treatment (CTR) and after a 24 or 48 h recovery time in the presence of fresh, complete medium. The results show the means of living cell number ± standard deviations (SD); *n* = 3. Solid-colored columns refer to the end of a 24 h treatment, colored and textured columns refer to a 24 h recovery time, and the colored and dotted columns refer to a 48 h recovery time. ** *p* < 0.01 vs. CTR cells at the end of a 24 h treatment; ^§§^ *p* < 0.01 vs. CTR cells after 24 h of recovery, and ^##^ *p* < 0.01 vs. CTR cells after a 48 h recovery time.

In order to evaluate the effects of CB on hASC viability and growth rate, cells were counted immediately after 24 h of CB treatment, and an additional 24 or 48 h after the chemical’s removal. The 24 h CB treatment reduced the number of living cells in treated samples compared to CTR samples (see solid-colored columns in Figure 3). Nevertheless, cell viability (obtained by calculating the percentage of living cells compared to the total number of counted cells) remained high even when the 10 µM CB concentration was used (Table 1).

Moreover, after CB wash-out, the number of hASCs increased over time in all investigated conditions (Figure 3), and cells retained high viability (Table 1). The effects elicited by CB were reversible, as demonstrated from cell counts obtained after 24 and 48 h recovery from CB withdrawal (textured and dotted columns in Figure 3, respectively).

We also calculated the hASC growth rate for each experimental condition (untreated cells; DMSO- and CB-treated cells) by comparing the number of cells after a 24 or 48 h recovery time with the corresponding number of cells at the end of the 24 h of treatment, as described in Section 2.5. Twenty-four hours after the CB removal, the growth rate of the CB-treated cells (0.1, 1, and 10 µM) was similar to that of CTR (CTR: 0.020 ± 0.013; DMSO: 0.023 ± 0.013; CB 0.1 µM: 0.026 ± 0.012; CB 1 µM: 0.025 ± 0.012; CB 10 µM: 0.022 ± 0.015). The same results were obtained 48 h after the CB wash-out (CTR: 0.021 ± 0.009; DMSO: 0.022 ± 0.012; CB 0.1 µM: 0.025 ± 0.013; CB 1 µM: 0.025 ± 0.012; CB 10 µM: 0.023 ± 0.014). Altogether, these data suggest that cells, after CB removal, began to divide again, retaining their proliferative capacity.

As in the metabolic assay, cells treated with 0.05% DMSO (the CB vehicle) showed no statistically significant differences in cell number and growth rate compared to CTR cells (Figure 3).

**Figure 4:**
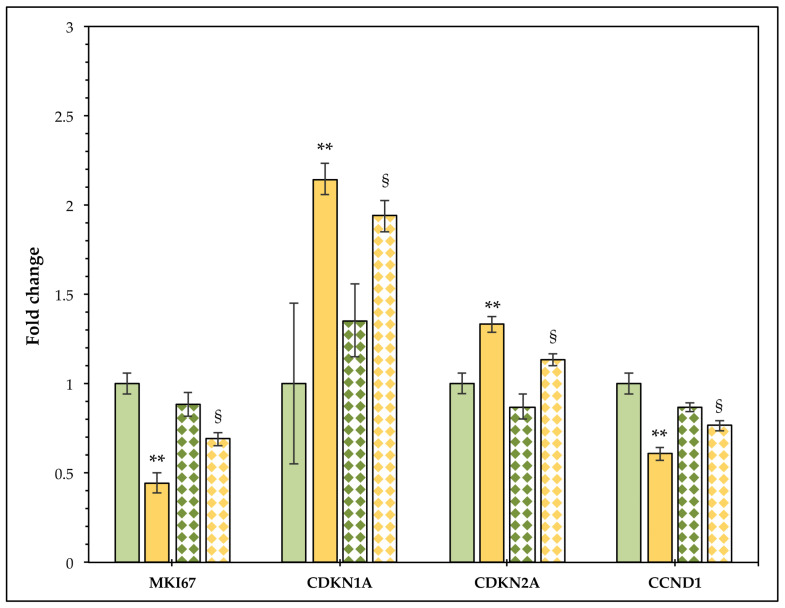
Gene expression in human adipose-derived stem cells (hASCs) after a 24 h cytochalasin B (CB) treatment and after 24 h of recovery time. hASC expression of *MKI67*, *CCND1*, *CDKN1A*, and *CDKN2A* genes was assessed after 24 h of treatment with CB 1 μM or without treatment (CTR) and 24 h after the chemical’s removal. Data were normalized using four reference genes (*GAPDH*, *RPL13*, *TBP*, and *HPRT1*); the normalized expression value of untreated cells (CTR) after a 24 h CB treatment was set to 1, and all other gene expression values were reported to CTR value. Graphic represents the normalized fold change ± standard error of mean (SEM); *n* = 3. Green and orange solid columns refer to CTR cells and to cells treated with CB 1 μM for 24 h, respectively; green and orange dotted columns refer to CTR cells and cells treated with CB 1 μM at the end of a 24 h recovery time, respectively. ** *p* < 0.01 vs. gene expression of CTR cells (green solid columns); ^§^ *p* < 0.05 vs. gene expression of treated cells at the end of a 24 h CB treatment (green dotted columns).

To confirm the effects of CB on cell cycle arrest and on cell proliferation block, the expression levels of markers involved in cell proliferation and cell cycle control, Ki-67, p21, p16^INK4α^, and cyclin D1, were investigated after treating cells for 24 h with CB 1 µM. CB reduced the expression of *MKI67* and *CCND1,* and increased the mRNA of *CDKN1A* (*alias p21*) and *CDKN2A* (*alias p16^INK4α^*) compared to the CTR (Figure 4, see solid columns). Moreover, after 24 h of recovery time, hASCs treated with CB 1 µM showed statistically significant differences in gene expression compared to the hASCs treated for 24 h with CB, confirming that cells were able to divide again, retaining their proliferative ability, in agreement with the cell count data.

### 3.4. Effects of Cytochalasin B on hASC Morphology and Metabolism

**Figure 5:**
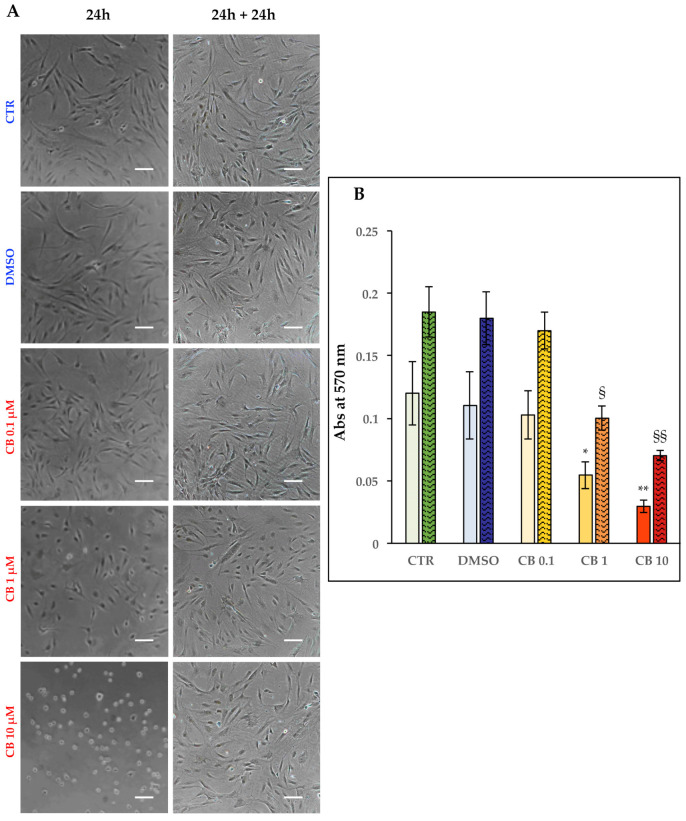
Morphology and metabolism of human adipose-derived stem cells (hASCs) after 24 h in the presence of different concentrations of cytochalasin B (CB), and after 24 h of recovery time. hASCs were seeded at 4000 cells/cm^2^ with CB 0.1, 1, and 10 μM, or 0.05% dimethyl sulfoxide (DMSO) (CB vehicle), or without treatment (CTR). (**A**) Representative images of hASCs at the end of the treatment and 24 h after the end of the treatment. Cells were detected under bright field illumination with a Nikon inverted microscope Eclipse TS100 and a digital sight camera DS-Fi1. Scale bars: 50 μm. (**B**) Columns related to cell metabolism represent the mean absorbances (Abs) at 570 nm of formazan salts at the investigated time points ± standard deviation (SD); *n* = 3. For each condition (treated or untreated cells), solid-colored columns refer to cell metabolism at the end of a 24 h treatment, and colored and textured columns refer to a 24 h recovery time. *n* = 3, * *p* < 0.05 and ** *p* < 0.01 vs. CTR cells; ^§^ *p* < 0.05 and ^§§^ *p* < 0.01 vs. CTR cells 24 h after the chemical wash-out.

In order to analyze the effects of CB on the cell morphology, hASCs were imaged after 24 h of exposure to CB (0.1, 1, and 10 μM) or 0.05% DMSO (the CB vehicle). DMSO treatment did not influence cell morphology (Figure 5A; see DMSO-treated vs. untreated cells, CTR). At the same time, CB induced alterations of cell morphology in a manner directly proportional to the CB concentrations. At 10 μM, the cells experienced a total collapse of their cytoplasms, despite still retaining the feature of viability to remain well adhered to the support provided by the tissue culture plastic. The dose-dependent effects on cell morphology persisted after 24 h of recovery time from the chemical, although the tendency of cells to regain their morphology was evident even in cells previously treated with the highest CB concentration (Figure 5A).

Concomitant with the morphological analysis, hASC metabolism was assessed with the colorimetric MTT assay. Treatments with CB resulted in a dose-dependent reduction in cell metabolism, when compared to control, untreated cells (CTR), both after a 24 h treatment and 24 h after the chemical wash-out (Figure 5B). In particular, cells treated with the two highest concentrations of CB showed a statistically significant reduction in metabolism (Figure 5B). Moreover, the removal of CB treatments allowed hASCs to increase their metabolic activity, without, however, reaching the levels of CTR cells, in agreement with the cell count and growth rate above data. On the other hand, dimethyl sulfoxide (DMSO)-treated cells showed no differences in cell metabolism compared to CTR (Figure 5B).

### 3.5. Live Cell Imaging of hASCs Treated with Cytochalasin B

The effect of CB on cell morphology was also evaluated using the time-lapse live cell imaging technique, which allows to study the change over time of the morphology of a single cell or a group of cells. The acquired images covered a 7 h period, and were used to obtain 10 s movies for each experimental condition (hASCs treated with CB 0.1, 1, or 10 μM or untreated, CTR) (Supplementary Movies S1–S4). The acquired images included both those collected from the exposed cells, and those obtained after treatment removal in order to monitor the cell morphology retrieval. Overall, CB withdrawal was performed by replacement with a CB-free medium as soon as a change in morphology was clearly evident, to allow observation of morphological recovery during the remaining time window. The time points for the toxin wash-out were dose-dependent. Thus, the removal of the highest CB concentration (10 μM) was executed when the CB exposure reached 1/3 of the overall observational window (7 h), a time point at which the effect of the molecule on cell morphology was already evident. Instead, to gain enough changes in cell morphology when CB 1 μM was used, the toxin was removed at 2/3 of the observational period. On the contrary, no evident morphological changes occurred during the 7 h time window when hASCs were treated with the lowest CB concentration of 0.1 μM. The acquired data confirmed cell morphology alterations in a dose-dependent manner (Supplementary Movies S1–S4). In fact, cells treated with a concentration of 10 μM CB appeared thinned and collapsed due to the action of CB on the cytoskeleton, compared to CTR cells. On the contrary, these alterations were less pronounced in cells treated with 1 μM CB and even less in those treated with 0.1 μM CB within the investigated period (Supplementary Movies S1–S4). Moreover, when CB was removed from the medium, cells tended to recover their morphology and to regain the elongated shape typical of hASCs (Supplementary Movies S1–S4).

### 3.6. Effects of Cytochalasin B on the Localization and Expression of Cytoskeletal Markers in hASCs

**Figure 6:**
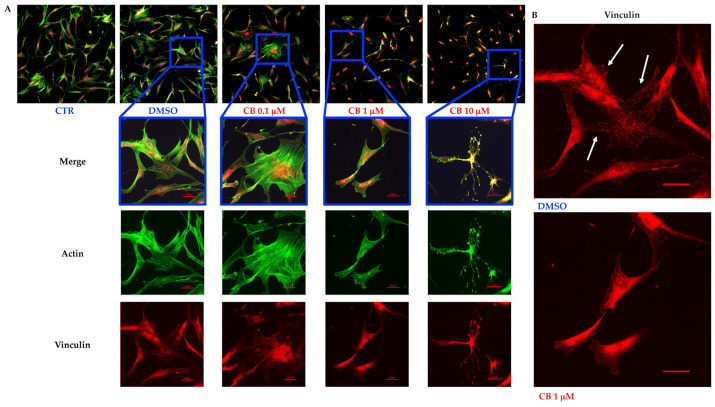
Immunofluorescence of human adipose-derived stem cells’ (hASCs) cytoskeletal markers after 24 h of cytochalasin B (CB) treatment. (**A**) hASCs were immunostained with Phalloidin (green signal, specific for F-actin) and anti-vinculin (red signal) after 24 h of 0.05% dimethyl sulfoxide (DMSO) (CB vehicle) or CB exposure (0.1, 1, or 10 μM). Untreated cells were used as the CTR. Acquisitions of images were performed with a Nikon inverted microscope Eclipse Ti2-E and a digital sight camera DS-Qi2, through the imaging software NIS-Elements. (**B**) Enlarged images referring to cells treated with 0.05% DMSO or CB 1 μM for 24 h. White arrows indicate some focal adhesions. Scale bars: 50 μm.

Untreated hASCs and hASCs incubated with 0.1, 1, or 10 μM CB, or 0.05% DMSO (the CB vehicle), were immunostained with Phalloidin (useful to visualize F-actin) and anti-vinculin after 24 h of exposure. As shown in Figure 6A (top), the hASC shape and the organization of cytoskeletal markers were similar in untreated and DMSO-treated cells. hASCs appeared elongated and well spread with actin fibers (green signal) aligned along the long axis of the cells. On the contrary, CB treatment induced a dose-dependent change in hASC shape and in actin organization. In particular, hASCs thinned and shrank, while the actin cytoskeleton progressively collapsed (Figure 6A). These changes were more evident after 3 days of treatment with CB, during which actin polymerization was blocked in a dose-dependent manner and CB-treatment resulted in the shortening and cytoplasmic punctuate of actin fiber organization (Supplementary Figure S2).

At the same time, vinculin (red signal), a membrane-cytoskeletal protein of focal adhesions, was expressed after CB treatment at each concentration, but the treatments modified the protein distribution at the subcellular level in a dose-dependent manner (Figure 6A and Supplementary Figure S2). In fact, the focal adhesions—well visible spots (in red, highlighted by white arrows in Figure 6B) in the presence of DMSO and 0.1 μM CB—were less evident at higher concentrations, indicating that the inhibition of actin polymerization entailed a global dysregulation in the macromolecular patterning within FACs, including the molecular transport inside the cells (Figure 6 and Supplementary Figure S2).

**Figure 7:**
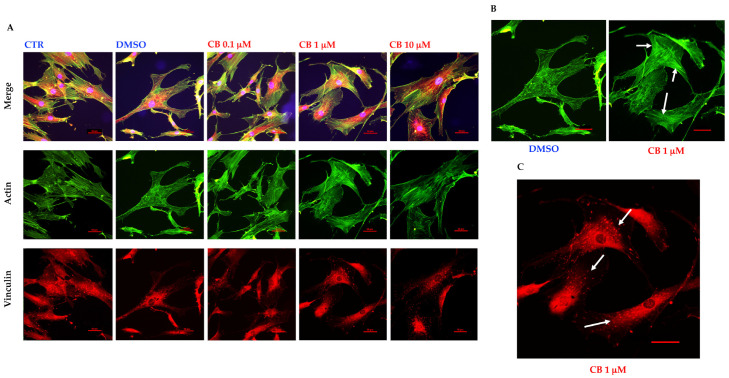
Immunofluorescence of human adipose-derived stem cells’ (hASCs) cytoskeletal markers after 24 h of recovery time from the cytochalasin B (CB) treatment. (**A**) hASCs were immunostained with Phalloidin (green signal, specific for F-actin) and anti-vinculin (red signal) 24 h after the treatment wash-out. Acquisitions of images were made with a Nikon inverted microscope Eclipse Ti2-E and a digital sight camera DS-Qi2, through the imaging software NIS-Elements. (**B**) Enlarged images referring to cells previously treated for 24 h with 0.05% dimethyl sulfoxide (DMSO) (CB vehicle) or CB 1 μM, after 24 h of recovery time. White arrows indicate the bundle of actin. (**C**) Enlarged images referring to cells previously treated for 24 h with CB 1 μM, after 24 h of recovery time. White arrows indicate some focal adhesions. Scale bars: 50 μm.

The immunostainings of cytoskeletal markers were also performed 24 h after the CB removal, and results are shown in Figure 7. In agreement with literature, the effect of CB on F-actin was reversible, and the microfilament organization (green signal) turned out to be similar in CB- and DMSO-pretreated hASCs, although greater bundle thickness was evident within mycotoxin-treated cells (Figure 7A,B). At the same time, after the CB removal, hASCs recovered their fibroblast-like shape. Vinculin (red signal) and focal adhesion patterns were completely restored and often colocalized with the ends of actin filaments (white arrows in Figure 7C).

### 3.7. Effects of Cytochalasin B on the Mechanical Properties of hASCs

The effects produced by CB on the cytoskeleton and consequently on the mechanical properties of hASCs were evaluated with AFM, which allows studying at the nano-scale level the changes in elasticity and viscosity in single cells. Firstly, the cells were analyzed using the force-volume method to produce a set of stiffness maps related to different experimental times. The elasticity values are expressed in terms of Young’s modulus (*E*_0_), calculated using the Hertz–Sneddon model while assuming a conical tip [57]. The control experiment, in which just 0.05% DMSO was used, produced no visible changes in the elastic behavior of cells. In the case of the low concentration of 1 μM CB, we observed a small decrease in the *E*_0_ values, whereas the treatment with 5 μM CB led to a notable decline in the *E*_0_ values (highlighted by darker color), and therefore in the cell stiffness, which persisted till the end of the analysis (Supplementary Figure S3). However, the analysis of the force curves according to the Hertz–Sneddon model completely neglected the time-dependent behavior of the sample reaction to the indenting tip. Viscosity changes that might happen upon cell drug injection were therefore not included in the analysis.

To obtain the complete viscoelastic characterization of cells treated with all the investigated CB concentrations (0.1, 1, 5, or 10 μM CB) and with DMSO, we applied Ting’s model associated with the power-law-relaxation (PLR) process to the approach/retract profiles of the force curves [58]. In this model, viscosity is represented by the α power-law exponent (α = 0 → completely elastic behavior; α = 1 → completely fluid behavior), whereas the cell elastic stiffness component is given by *E*_0_—i.e., the initial Young’s modulus felt by the cantilever tip upon contact with the sample. We started with the CTR cells to analyze cell rheological stability in the measurement conditions. This aspect is particularly relevant, because in AFM experiments we can keep the temperature at 37 °C, but we cannot keep the CO_2_ concentration at 5% as typically requested by cell culture conditions. As a consequence, total measurement time was kept within 2 h after cells were inserted in the AFM set-up.

**Figure 8:**
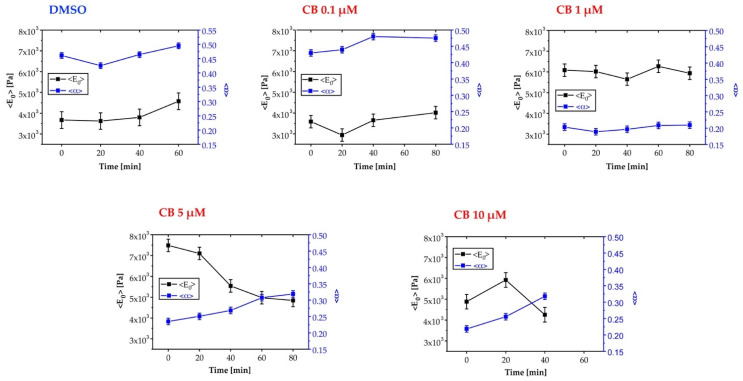
Evaluation of changes in human adipose-derived stem cells’ (hASCs) viscoelasticity during the treatment with different concentrations of cytochalasin B (CB) using Ting’s model. Time-wise behavior for the representative Young’s modulus (*E*_0_) and power-law exponent (α) values in the control (dimethyl sulfoxide, DMSO, 0.05%) and 0.1, 1, 5, and 10 μM CB cases. The continuous lines are guides for the eye.

As shown in Figure 8, results obtained by the analysis of stiffness and viscosity maps using Ting’s model revealed that for cells treated with DMSO (control sample of the experiments), a small increase in the *E*_0_ elastic modulus occurred after about 40 min, whereas for the viscosity related parameter (α), an almost constant value was observed. Upon exposure to CB, in the cases of 0.1 and 1 μM we observed an initial small decrease in *E*_0_, followed by an inversion of the trend which took the *E*_0_ parameter to a value comparable with the initial one (Figure 8). We interpret this behavior as being due to the initial effect of CB, followed by the prevalence of the typical behavior observed in control experiments. For the treatment with 5 μM CB, we observed a continuous decrease in *E*_0_ values, and therefore, in the instantaneous stiffness of hASCs: the values of α increased, suggesting that the cells became more liquid-like, as compared with the control conditions. In the case of 10 μM CB, the rheological analysis is difficult because cells underwent an immediate morphological rearrangement, as is also evident from time-lapse experiments. By exploiting Ting’s model, we obtained oscillating behavior for the *E*_0_ value and a tendency to increase for the viscosity parameter (Figure 8).

**Figure 9:**
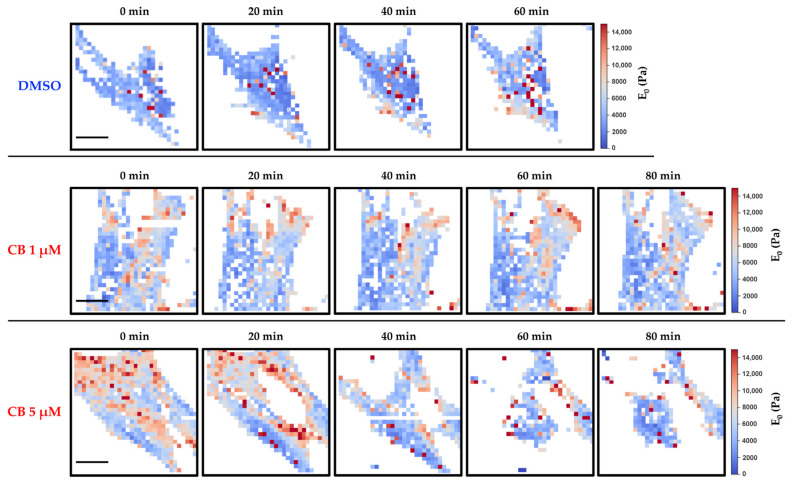
Evaluation of changes in human adipose-derived stem cells’ (hASCs) stiffness during the treatment with different concentrations of cytochalasin B (CB) using Ting’s model. hASCs were analyzed with the atomic force microscope (AFM) for a total time of about 2 h. Images are representative stiffness maps of single cells (seeded in a Petri dish at density of 4000 cells/cm^2^) at different times, treated with 0.05% dimethyl sulfoxide (DMSO) (CB vehicle) or CB at 1 or 5 μM. The specific value of the Young’s modulus (*E*_0_) at each point is represented by a color from a scale. The corresponding scale is reported on the right for each line. The white regions around the cell represent the substrate. Scale bars: 20 μm.

**Figure 10:**
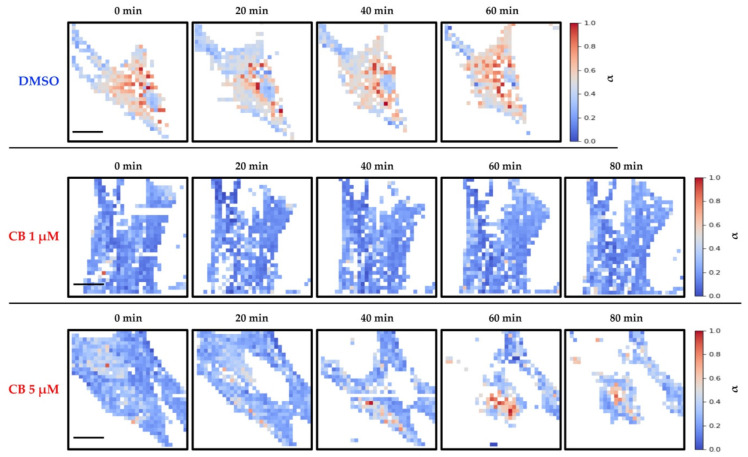
Evaluation of changes in human adipose-derived stem cells’ (hASCs) viscosity during the treatment with different concentrations of cytochalasin B (CB) using Ting’s model. hASCs were analyzed with the atomic force microscope (AFM) for a total time of about 2 h. Images represent maps of the viscosity parameter α of single cells (seeded in a Petri dish at density of 4000 cells/cm^2^) at different times treated with 0.05% dimethyl sulfoxide (DMSO) (CB vehicle) or CB at 1 or 5 μM. Each point in a map refers to a specific value of the power-law exponent (α) and refers to a color scale. The color scale is reported on the right of each line. The white regions around the cell represent the substrate. Scale bars: 20 μm.

In Figure 9 and Figure 10, representative stiffness and viscosity maps of single cells (treated with DMSO or 1 and 5 μM CB) are shown, respectively. Red points indicate higher *E*_0_ or α values, and blue points indicate lower values.

### 3.8. Effects of Cytochalasin B on the Adipogenic Ability of hASCs

Before starting the adipogenic induction protocol, the effects of CB on hASC viability were evaluated for up to 14 days of treatment using all investigated concentrations (0.1, 1, 5, and 10 μM). Viability was analyzed 3, 6, and 14 days from the beginning of CB treatment. It was >85% even for the highest concentration of CB, 10 μM (see Supplementary Table S1).

**Figure 11:**
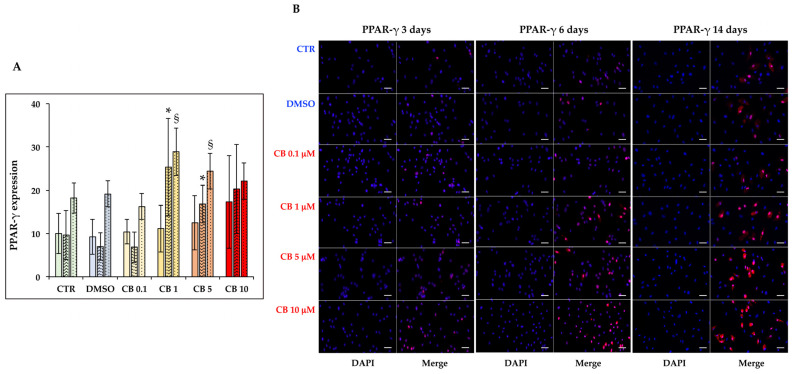
Immunofluorescence of peroxisome proliferator-activated receptor gamma (PPAR-γ) after 3, 6, and 14 days of induced differentiation in the presence of cytochalasin B. PPAR-γ (red signal) staining was performed in hASC cultures after 3, 6, or 14 days of adipogenic induction in the absence or presence of CB (0.1, 1, 5 and 10 μM) or 0.05% dimethyl sulfoxide (DMSO) (CB vehicle). (**A**) PPAR-γ expression after 3 (solid-colored columns), 6 (textured, colored columns), and 14 (dotted, colored columns) days of adipogenic differentiation. Graph shows the mean percentages of PPAR-γ positive cells ± standard deviations (SD), *n* = 3, * *p* < 0.05 vs. CTR at 6 days; ^§^ *p* < 0.05 vs. CTR at 14 days. (**B**) Representative images of PPAR-γ (red signal) staining after 3, 6, and 14 days of adipogenic induction. Nuclei were stained with NucBlue^TM^ (DAPI). Acquisitions of images were made with a Nikon inverted microscope Eclipse Ti2-E and a digital sight camera DS-Qi2, through the imaging software NIS-Elements. Scale bars: 50 μm.

**Figure 12:**
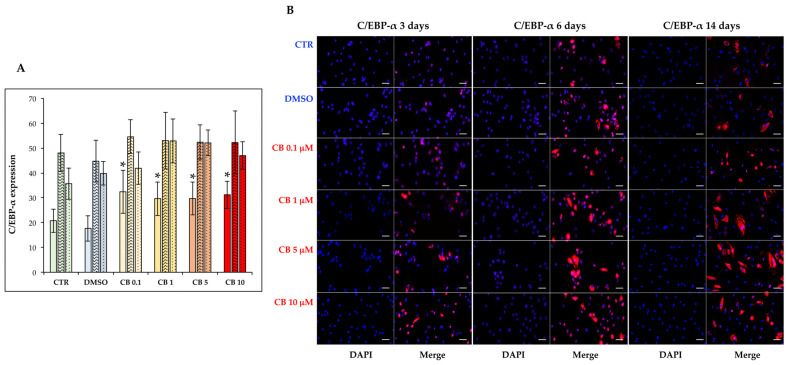
Immunofluorescence of CCAAT/enhancer-binding protein alpha (C/EBP-α) after 3, 6, and 14 days of induced differentiation in the presence of cytochalasin B. C/EBP-α (red signal) staining was performed in hASC cultures after 3, 6, or 14 days of adipogenic induction in the absence or presence of CB (0.1, 1, 5 and 10 μM) or 0.05% dimethyl sulfoxide (DMSO) (CB vehicle). (**A**) C/EBP-α expression at 3 (solid-colored columns), 6 (textured, colored columns), and 14 (dotted, colored columns) days of adipogenic differentiation. Graph shows the mean percentages of C/EBP-α-positive cells ± standard deviations (SD); *n* = 3; * *p* < 0.05 vs. CTR at 3 days. (**B**) C/EBP-α (red signal) staining was performed in hASC cultures after 3, 6, and 14 days of adipogenic induction. Nuclei were stained with NucBlue^TM^ (DAPI). Acquisitions of images were made with a Nikon inverted microscope Eclipse Ti2-E and a digital sight camera DS-Qi2, through the imaging software NIS-Elements. Scale bars: 50 μm.

Then, the effects of CB on hASC adipogenic commitment were studied by adding CB in the presence of the induction medium for the entire time of differentiation. The adipogenesis was monitored by evaluating the expression and localization of two adipogenic markers, PPAR-γ and C/EBP-α, and by counting the numbers of perilipin and O.R.O-positive cells. The expression and localization of PPAR-γ and C/EBP-α were assessed by immunofluorescence 3, 6, and 14 days after the beginning of treatment (Figure 11 and Figure 12). The percentages of PPAR-γ and C/EBP-α positive cells were calculated and are reported in Figure 11A and Figure 12A, respectively. For both markers, no difference was observed between untreated and DMSO (0.05%) treated cells, but CB influenced their expression. In particular, CB at 1 and 5 µM increased the expression of both adipogenic markers statistically significantly (*p* < 0.05)—for C/EBP-α after 3 days and for PPAR-γ after 6 and 14 days—suggesting that the presence of these CB concentrations in the differentiation medium stimulated adipogenic differentiation.

**Figure 13:**
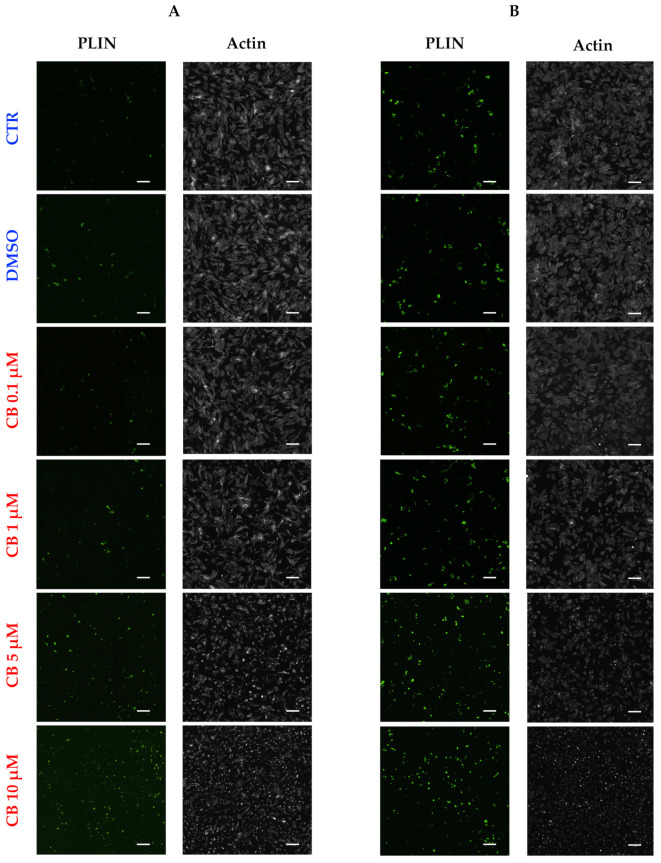
Adipogenic potential of human adipose-derived stem cells (hASCs) in the presence of cytochalasin B (CB). hASCs were induced to adipogenesis for 3 and 6 days with the StemPro^®^ Adipogenesis Differentiation medium, supplemented with 0.05% dimethyl sulfoxide (DMSO) (CB vehicle) or CB at 0.1, 1, 5 or 10 μM. Cells cultured only with the StemPro^®^ Adipogenesis Differentiation medium were used as a CTR. The progression of the adipogenic commitment was assessed by evaluating the expression of perilipin (PLIN, green signal) and by immunostaining with Phalloidin (grey signal, specific for F-actin). Images were acquired by the Nikon inverted microscope Eclipse Ti2-E and a digital sight camera DS-Qi2, through the imaging software NIS-Elements. (**A**) Images taken 3 days after the beginning of the adipogenic protocol. (**B**) Images taken 6 days after the beginning of the adipogenic protocol. Scale bars: 100 μm.

Moreover, the expression of perilipin, a protein associated with the surface of lipid droplets, was investigated to evaluate the effects of CB on the progression of adipogenic commitment after 3 and 6 days of the differentiation protocol. As shown in Figure 13, perilipin expression increased in a dose- and time-dependent manner in hASCs treated with CB when compared to CTR cells. Over 40% of cells were perilipin-positive after being treated for 6 days with the highest concentrations of CB (Table 2). At the same time, the organization of the actin cytoskeleton, and consequently, the hASC shape, were strongly modified with the higher CB concentrations (Figure 13 and Supplementary Figure S4).

**Figure 14:**
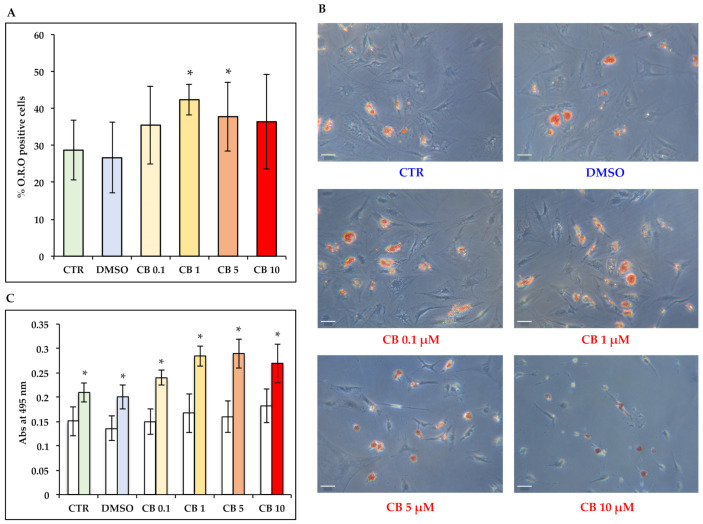
Adipogenic potential of human adipose-derived stem cells (hASCs) in the presence of cytochalasin B (CB). hASCs were induced to adipogenesis for a total period of 14 days with the StemPro^®^ Adipogenesis Differentiation medium, supplemented with 0.05% dimethyl sulfoxide (DMSO) (CB vehicle) or CB at 0.1, 1, 5, or 10 μM. Cells cultured only with the StemPro^®^ Adipogenesis Differentiation medium were used as a CTR. The final adipogenic commitment was evaluated by Oil Red O (O.R.O) staining. (**A**) Undifferentiated unstained and O.R.O-stained cells were counted using ImageJ software. The percentage of O.R.O positive cells was calculated. Graph shows the mean percentages of O.R.O positive cells ± standard deviations (SD); *n* = 3; * *p* < 0.05 vs. CTR. (**B**) Representative O.R.O images acquired under phase contrast illumination with the Leica MC170 HD Imaging System. Cells positive for adipogenesis showed red vacuoles in the cytoplasm. Scale bars: 50 μm. (**C**) White histograms represent data derived from hASCs cultured in basal medium, and colored histograms represent those from hASCs treated with adipogenic medium. The lipid-rich vacuoles detected by O.R.O dye were extracted, and their absorbance was read at 495 nm with a spectrophotometer. Data are expressed as mean of lipid content at 495 nm absorbance (Abs) ± SD. The * represents the significance of differences between data obtained from hASCs cultured in basal medium and hASCs cultured in adipogenic medium (* *p* < 0.05, *n* = 3).

Moreover, at the end of the 14 day differentiation protocol involving staining cells with the O.R.O solution, which colored in red the lipid vacuoles present in differentiated cells, we found that CB influenced the final adipogenic commitment of hASCs, while modulating the organization of the cytoskeletal actin (Figure 14 and Supplementary Figure S5). In particular, at 1 and 5 μM, CB enhanced the hASC adipogenic potential in a statistically significant manner compared to CTR and DMSO-treated cells (Figure 14A).

Noteworthy, in hASCs cultured without the induction medium, we found a slight increase in the expression of PPAR-γ at the highest concentrations of CB compared to standard medium, without, however, effectively promoting the adipogenic commitment (data not shown). These data were also confirmed from the reading of the absorbance at 495 nm after the extraction of O.R.O staining from each sample. In fact, lower absorbance values for cells cultured only in standard medium with CB supplementation with respect to induced cells demonstrated first the efficiency of the adipogenic induction protocol, but also the inability of CB to act alone in the adipogenic program (Figure 14C).

## 4. Discussion

Actin microfilaments, together with microtubules and intermediate filaments, are essential components of the cytoskeleton, and appear as helical structures approximately 7 nm in diameter, with a pitch of about 37 nm. Besides transducing mechanical stimuli, actin filaments are highly electrostatically charged [59,60]. Thus, the head-to-head arrangement of actin monomers into dimeric structures produces an alternate patterning of electric dipole moments along the filament, establishing a sort of intracellular bioelectronic circuit [59,61].

Agents interfering with the structure and polymerization dynamics of actin filaments are expected to affect the mechano-sensing and mechano-transduction of the cells, thereby influencing normal morphogenetic decisions and precisely controlling stem cell fate, and resulting in larger-scale anatomy [62,63,64,65,66].

Consistent with these observations, the CB factor, which inhibits actin polymerization, has been shown to interfere with complex cellular processes, such as the transfer of human artificial chromosomes or of mitochondria from donor to recipient cells [67,68,69], and to influence the abilities of hMSCs to proliferate and differentiate into specific lineages [11]. Moreover, different cytochalasins have been used to explore the roles of the actin component in the mechanical properties of cells, as probed by AFM [70,71,72,73]. Typically, these investigations initially considered only elastic behavior of cells when indented by the cantilever tip, and in several cases a reduction in the cell stiffness was found following exposure of cells to cytochalasin. Using different strategies, including the exploitation of Ting’s model, the possibility of a complete rheological characterization of living cells by AFM emerged [74,75]. These studies also investigated the effects of cytochalasins, prevalently focused on CD, and reported a strong effect of cytochalasins on the viscous properties of the cells.

In the current study, we investigated for the first time the effects of CB on nanomechanical properties, and consequently, on several characteristics of a specific type of hMSCs isolated from adipose tissue, the hASCs. These cells are suitable candidates for regenerative medicine, owing to their easy and minimally invasive harvesting, higher abundance in their tissue source compared to the bone marrow, and wide range of differentiation pathways and paracrine repertoire [42]. Our AFM investigation showed that CB strongly affects the viscosity of hASCs, shifting their behavior towards that of a pure liquid material (an increase in α), whereas the elastic component (*E*_0_) is little affected at low concentrations. In fact, we observed a continuous decreasing trend with time of the *E*_0_ parameter only with CB at 5 μM. At the same time, we found an increase in the viscosity of cells exposed to CB. The variation of the viscosity we observed was probably due to a fragmentation of the actin cytoskeleton and to an increase in the actin fragment density inside the cell cytoplasm, leading to a higher G-actin/F-actin ratio, thereby producing an increased ability of the cytoplasm to flow in the presence of an applied force.

As matter of fact, CB treatment induced a dose-dependent destruction of the cytoskeleton, as demonstrated by Phalloidin staining, and by the altered distribution of vinculin associated with FAC disappearance in response to the higher CB concentrations. Similar effects on the cytoskeleton were shown in other stem cell models, such as hBM-MSCs, and also with CD [76,77,78,79].

Our AFM and cytoskeletal data provide the nanomechanical cues for interpreting the reversible and dose-dependent actions exerted by CB on hASC morphology, proliferation, metabolic activity, and differentiation. In fact, in association with the cytoskeletal alterations, hASCs treated with CB changed their morphology, as was evident with microscopic and live cell imaging analyses—losing their typical fibroblast-like shape in a dose-dependent manner.

Moreover, CB-induced cytoskeletal changes affected the proliferative ability of hASCs, resulting in a lower cell number and lower metabolic activity after 24 h of CB treatment. At the same time, even at the highest concentrations of CB, the viability of the hASCs was not particularly affected, and after CB’s removal from the culture medium, the cells showed a proliferative rate similar to control cells. Some studies demonstrated that CD or CB did not compromise cell proliferation or viability [40,80], but on the contrary, other research reported decreases in both parameters dose- and treatment time- dependently [81,82,83]. In particular, Kocsis and colleagues (2021) showed a great difference in cell viability between MSCs treated with CB for 3 or 24 h, with low (1–8 μM) or high concentrations of this chemical (until 128 μM) [82]. However, for the range of CB concentrations used in our studies, they found a reduction in viability after 24 h of treatment comparable to or slightly greater than our results.

Since CB treatment only slightly affected cell viability, the low metabolic/proliferative capacity observed in 1 and 10 μM CB treated-cells could be mainly due to the arrest of the cell cycle.

The effects of CB on the cell cycle progression were confirmed by the gene expression analysis of the proliferative marker MKI67 and of the cell cycle regulators p21, p16^INK4α^, and cyclin D1. While *CDKN1A* (*alias p21*) and *CDKN2A* (*alias p16^INK4α^*) gene expression increased, the amounts of *CCND1* and *MKI67* mRNA decreased in hASCs treated with CB for 24 h, compared to CTR cells. p21 and p16^INK4α^, proteins activated by external stressors, such as chemicals and stress culture [84], inhibit the formation of the CDK–cyclin complexes, thereby arresting the cell cycle during the transition between G1 and S phases, and preventing cell proliferation. In particular, p16^INK4α^ prevents the CDK4–cyclin D1 complex’s formation [85,86]. Moreover, when CB was removed from the culture medium, the expression of these genes tended to return to the level of the untreated cells. These results were in accordance with the grow rate data, which showed that hASCs were able to divide again and to partially recover their metabolic activity, mainly due to the restoration of cytoskeletal organization and morphology, following CB removal.

The CB-induced nanomechanical changes, associated with actin depolymerization, could also explain the significant increase in adipogenesis observed when hASCs were induced to differentiate. In fact, the CB-dependent cytoskeletal collapse correlated with the increase in perilipin expression and the increase in lipidic vacuoles in treated cells compared to CTR cells. In addition, the changed expression levels of the two adipogenic players PPAR-γ and C/EBP-α 3, 6, and 14 days into the CB exposure, indicate that CB can be exploited to finely and timely tune actin filament dynamics to afford selected manipulation of tissue-restricted genes in hASC differentiation. Noteworthy, the effects of CB on the cytoskeleton could explain the increase in PPAR-γ even when cells are cultured without the induction medium. However, additional factors may be required to complete the adipogenic program. PPAR-γ, an early adipogenic marker, is strongly expressed in the first days of differentiation, and it induces the expression of C/EBP-α. Both proteins act synergically to promote the expression of other adipocyte genes. On the other hand, C/EBP-α maintains the expression of PPAR-γ through a positive feedback loop that could justify the oscillatory expression of these two genes that we observed during the adipogenic program [87,88]. CB’s ability to promote adipogenesis when it was added to adipogenic medium during the entire induction protocol was also demonstrated when CD factor was used [35,78]. Similar effects were also observed in hBM-MSCs when CD was added to the medium each time it was changed during the adipogenic protocol [79], or only in a 24 h pretreatment [77].

Therefore, our data collectively demonstrated that the differentiating effects of CB occurred within timely and concentration-dependent modification profiles of the viscoelastic properties of the hASCs This encourages further deployment of AFM-executed analysis of CB action in future studies aiming at discovering the nanomechanical effects on the actin cytoskeleton with selective exposure times and concentrations. This perspective may set the basis for the exploitation of targeted, CB-induced nanomechanical changes capable of directing the stem cell fate other than the currently observed adipogenic lineage. In particular, the effects of CB on the spatial and molecular interactions among actin, intermediate filaments, and microtubules remain to be clarified. In fact, we hypothesize that these cytoskeletal proteins influence each other, as shown by Fan and collaborators (2020) in hASCs during osteogenesis, where the amounts and the spatial positions of vimentin and actin stress fibers exhibited opposite trends [34]. At the same time, it would be interesting to deepen the role of cofilin or destrin, a family of actin-binding proteins associated with the rapid depolymerization of actin microfilaments, alone or in association with CB treatment. Chen and colleagues (2018) have previously reported that enhancing F-actin stabilization through siRNAs mediated decreased levels of cofilin and destrin, inhibiting adipocyte differentiation, in hBM-MSCs [77].

Finally, a number of interrelated observations showed that an increase in cytoskeletal stiffness underlies: (i) the senescence process observed in MSCs isolated from the Zmpste24 mouse model of Hutchinson–Gilford progeria syndrome [89]; (ii) the aged profile observed in tendon/stem progenitor cells (TSPC) from elderly patients, which protract the time for the repair of tendon injuries [90], and in the fibrogenic conversion of muscle stem cells during age-related decline muscle regeneration [91]. Whether AFM analysis may help with defining specific conditions for CB treatment to counteract stem cell senescence and modulate the multilineage potential of hASCs remains to be established. Similarly, establishing whether CB-mediated responses may entail changes in the nuclear or cell surface level activity of mechano-sensors (such as the Yes-associated protein/transcriptional coactivator with PDZ-binding motif—YAP/TAZ, or integrin molecules, respectively) will be an interesting field of enquiry. Clarification of these issues must await further functional and molecular approaches and is a subject for our future investigations.

## 5. Conclusions

In conclusion, we showed for the first time that CB acted at the nanomechanical level to modulate hASC stiffness and viscosity, affording dose and time-dependent modulation of stem cell properties, including adipogenic ability. These observations set the basis for using CB as a potential and alternative tool for further elucidating and potentially controlling the mechano-sensing/mechano-transduction, as well as the differentiative ability of human stem cells, favoring their use in a therapeutic approach. To this end, autologous fat grafting is now a widely used approach in reconstructive and aesthetic surgical procedures, including the treatment of scar contractions, breast asymmetry, craniofacial abnormalities, and velo-pharyngeal insufficiency in cleft palate; and the treatment of radiation damage, breast capsular contracture, posttraumatic deformities, congenital abnormalities, and burn injuries. There is evidence that hASCs residing within the stromal vascular fraction of the adipose tissue, with their capability of differentiating into adipocytes, remarkably contribute to the process of graft survival after surgical implantation, and to the successful outcomes of plastic/reconstructive procedures [92]. Nevertheless, increased tissue stiffness, as occurs in cases of severe tissue damage, represents a major hurdle, often presenting the dual challenge of softening remodeled tissues subjected to scarring and achieving acceptable tissue augmentation in cases of loss-of-volume processes. Within this context, the ability of CB to enhance the adipogenic commitment in hASCs may prove rewarding in optimizing soft tissue augmentation, thereby decreasing the unpredictable resorption rates that have been reported to range from 20% to 90%, consequently severely reducing the total implanted volume [93,94]. Moreover, the proadipogenic action of CB, and its ability of finely tuning the viscoelastic properties in hASCs, may provide a population of hASC-derived adipocytes capable of counteracting tissue stiffness and remodeling not only on the body’s surface, but even at the level of the body’s interior, such as in intraabdominal adhesions that are among the most frequent pathological outcomes in general surgical practice, and result in increased morbidity and mortality [95].

## Figures and Tables

**Table 1:** Cell viability of human adipose-derived stem cells (hASCs) after 24 h cytochalasin B (CB) treatment and after a 24 or 48 h recovery time.

Group	*t* of Analysis 24 h	*t* of Analysis 24 h + 24 h	*t* of Analysis 4 h + 48 h
CTR	99.4 ± 1.5	97.8 ± 1.9	97.9 ± 2.1
DMSO	99.0 ± 2.4	96.1 ± 4.0	97.1 ± 2.7
CB 0.1 µM	100.0 ± 0.0	96.7 ± 2.8	97.0 ± 3.1
CB 1 µM	96.1 ± 4.9	98.4 ± 1.8	96.9 ± 2.8
CB 10 µM	89.1 ± 7.0	93.9 ± 5.8	94.1 ± 4.1

*t*: time; 24 h: after 24 h of treatment; 24 h + 24 h: 24 h after chemical wash-out; 24 h + 48 h: 48 h after chemical wash-out; CB: cytochalasin-B-treated cells; DMSO: dimethyl sulfoxide-treated cells; CTR: control (untreated cells). Cell viability: percentage of living cells/total number of cells ± standard deviation (SD).

**Table 2:** Perilipin expression in human adipose-derived stem cells (hASCs) after 6 days of adipogenic induction in the presence of cytochalasin B (CB).

Group	*t* of Analysis 6 Days
CTR	22.4% ± 5.3
DMSO	25.9% ± 3.6
CB 0.1 µM	30.9% ± 6.7
CB 1 µM	44.9% ± 9.1
CB 5 µM	42.8% ± 4.5
CB 10 µM	49.8% ± 9.8

*t*: time; 6 days: after 6 days of adipogenic induction in the absence or presence of cytochalasin B; CB: cytochalasin B-treated cells; DMSO: dimethyl sulfoxide-treated cells; CTR: control (untreated cells). Undifferentiated, unstained, and perilipin-positive stained cells were counted using the ImageJ software. Percentage of perilipin-positive cells was calculated. Table shows the mean percentages of perilipin-positive cells ± standard deviation (SD), *n* = 3.

## Data Availability

The datasets used and/or analyzed during the current study are available from the corresponding author on reasonable request.

## Supplementary Materials

The following data are available online at https://www.mdpi.com/article/10.3390/cells11101629/s1. Figure S1: Representative force curve with the approach and retracting portions. The graph shows the value of the applied force as a function of time, and in red is the fit of Ting’s model to obtain the Young’s modulus (*E*_0_) value and the power-law (α) exponent. Figure S2: Immunofluorescence of human adipose-derived stem cell (hASC) cytoskeletal markers after 3 days of cytochalasin B (CB) treatment. hASCs were immunostained with Phalloidin (green signal, specific for F-actin) or anti-vinculin (red signal) after 3 days in the absence or presence of CB (0.1, 1, or 10 μM). Acquisitions of images were performed with a Nikon inverted microscope Eclipse Ti2-E and a digital sight camera DS-Qi2, through the imaging software NIS-Elements. Figure S3: Evaluation of changes in human adipose-derived stem cells’ (hASCs) elasticity using the Hertz–Sneddon model during the treatment with different concentrations of cytochalasin B (CB). hASCs were evaluated with an atomic force microscope (AFM) for a total time of 2 h. Images are representative stiffness maps of single cells (seeded in a Petri dish at density of 4000 cells/cm^2^) at different experimental times and treated with (**A**) 0.05% dimethyl sulfoxide (DMSO) (CB vehicle), (**B**) CB at 1 μM and (**C**) CB at 5 μM. Each point in a map is associated with a specific value of Young’s modulus (*E*_0_) and is colored according to a color scale. The color scale is reported on the right of each line. The black regions around the cell represent the substrate. Scale bars: 20 μm. Figure S4. Adipogenic potential of human adipose-derived stem cells (hASCs) in the presence of cytochalasin B (CB). Adipogenesis was induced with hASCs for 3 days with the StemPro^®^ Adipogenesis Differentiation medium supplemented with CB at 1 and 5 μM. Cells cultured only with the StemPro^®^ Adipogenesis Differentiation medium were used as a CTR. The progression of the adipogenic commitment was assessed by immunostaining with Phalloidin (grey signal, specific for F-actin). Images were acquired by the Nikon inverted microscope Eclipse Ti2-E and a digital sight camera DS-Qi2, through the imaging software NIS-Elements, 3 days after the beginning of the adipogenic protocol; scale bars: 50 μm. Table S1: Cell viability of human adipose-derived stem cells (hASCs) after 3, 6, or 14 days of cytochalasin B (CB) treatment. *T*: time; 3, 6, or 14 days: after 3, 6, or 14 days of treatment; CB: cytochalasin-B-treated cells; DMSO: dimethyl-sulfoxide-treated cells; CTR: control (untreated cells). Cell viability: percentage of living cells/total number of cells ± standard deviation (SD). Figure S5: Immunofluorescence of human adipose-derived stem cell (hASC) cytoskeletal markers after 14 days of cytochalasin B (CB) treatment in the absence or presence of adipogenic medium. hASCs were immunostained with Phalloidin (grey signal, specific for F-actin) in the absence or presence of CB (0.1, 1, 5, and 10 μM). DMSO: dimethyl sulfoxide; induced: hASCs treated with adipogenic medium for 14 days; not induced: hASCs maintained in standard medium for 14 days. Acquisitions of images were made with a Nikon inverted microscope Eclipse Ti2-E and a digital sight camera DS-Qi2, through the imaging software NIS-Elements. Scale bars: 50 μm. Movie S1: Seven-hour live cell imaging of untreated hASCs (CTR). Movie S2: Seven-hour live cell imaging of hASCs treated with cytochalasin B (CB) at 0.1 μM. Movie S3: Seven-hour live cell imaging of hASCs treated with cytochalasin B (CB) at 1 μM for 2/3 of the analysis time. Movie S4: Seven-hour live cell imaging of hASCs treated with cytochalasin B (CB) at 10 μM for 1/3 of the analysis time.
