# Supplementary material for: Cytochalasin B Modulates Nanomechanical Patterning and Fate in Human Adipose-Derived Stem Cells
Source: Cells. 2022 May 12;11(10):1629. doi: 10.3390/cells11101629 (PMC9139657; doi:10.3390/cells11101629)
Supplement: Supplementary file 1 [file cells-11-01629-s001.zip › Supplementary Table S1.pdf]

Table S1. Cell viability of human adipose-derived stem cells (hASCs) after 3, 6 or 14 days of Cytochalasin B (CB) treatment.

Group	*t* of analysis<br><br>3 days	*t* of analysis<br><br>6 days	*t* of analysis<br><br>14 days
**CTR**	96.4 ± 1.1	94.2 ± 2.2	93.6 ± 1.4
**DMSO**	94.2 ± 3.2	93.8 ± 2.7	91.4 ± 3.5
**CB 0.1 μM**	94.7 ± 3.8	93.7 ± 2.3	92.3 ± 2.2
**CB 1 μM**	94.0 ± 3.4	93.3 ± 2.7	92.2 ± 3.3
**CB 5 μM**	92.8 ± 1.2	88.3 ± 3.3	87.2 ± 3.8
**CB 10 μM**	91.8 ± 2.8	88.0 ± 2.6	87.9 ± 2.0

t: time; 3, 6 or 14 days: after 3, 6 or 14 days of treatment; CB: Cytochalasin B treated cells; DMSO: dimethyl sulfoxide treated cells; CTR: control (untreated cells). Cell viability: percentage of living cells/total number of cells ± standard deviation (SD).
