# Supplementary figures and images for: Cytochalasin B Modulates Nanomechanical Patterning and Fate in Human Adipose-Derived Stem Cells
Source: Cells. 2022 May 12;11(10):1629. doi: 10.3390/cells11101629 (PMC9139657; doi:10.3390/cells11101629)

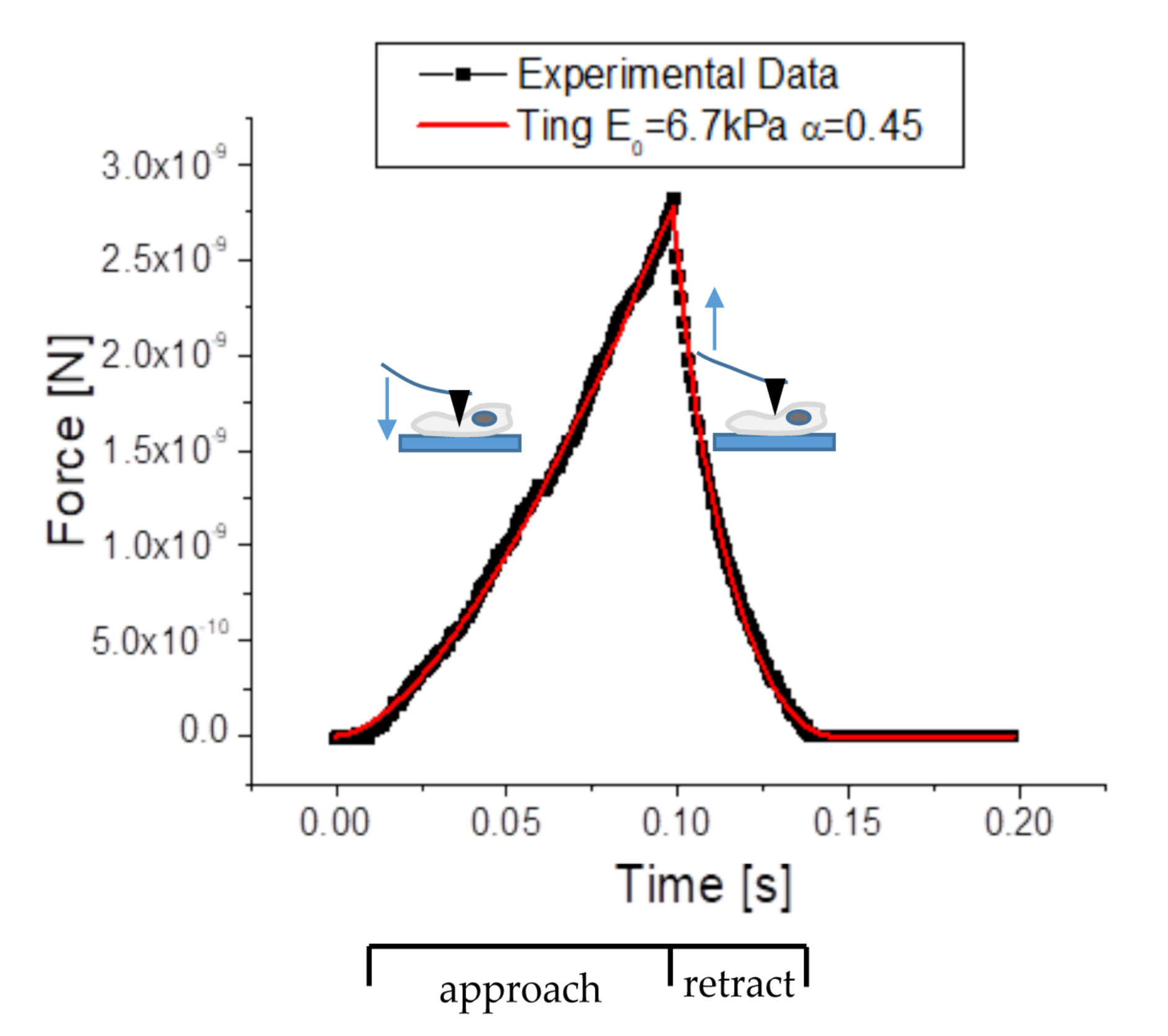

Supplement: Supplementary file 1 [file cells-11-01629-s001.zip › Supplementary Figure S1.jpg]

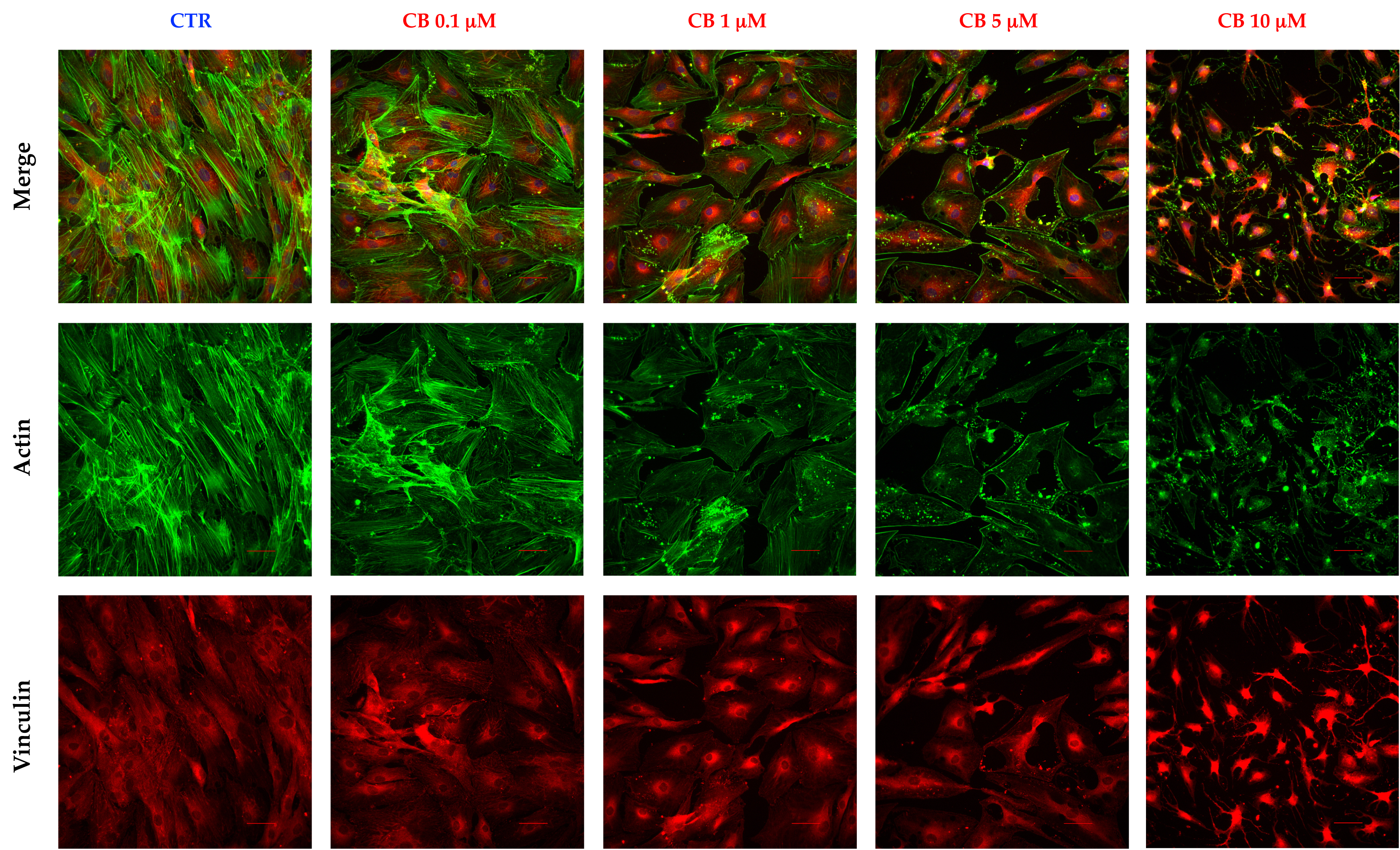

Supplement: Supplementary file 1 [file cells-11-01629-s001.zip › Supplementary Figure S2.jpg]

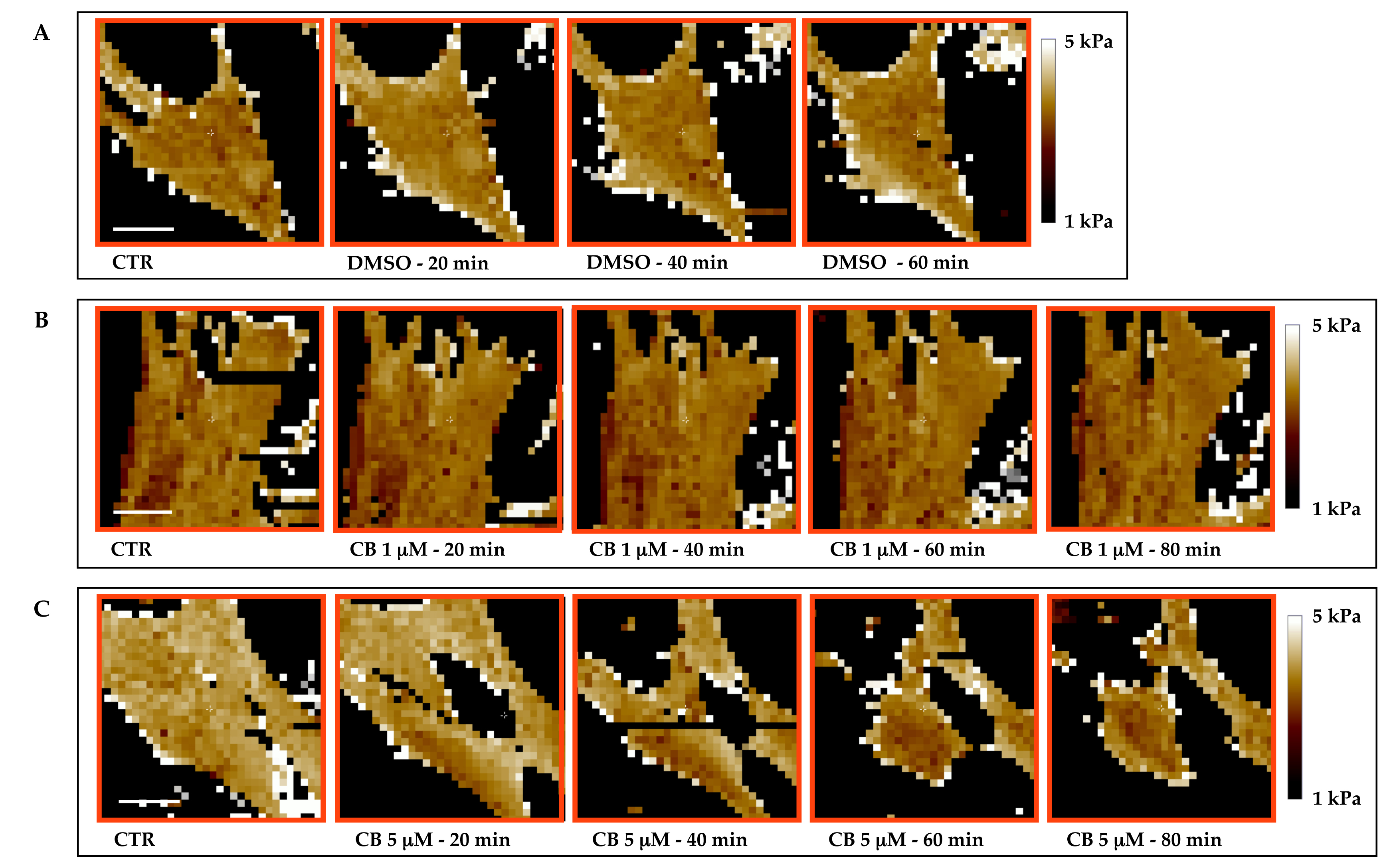

Supplement: Supplementary file 1 [file cells-11-01629-s001.zip › Supplementary Figure S3.jpg]

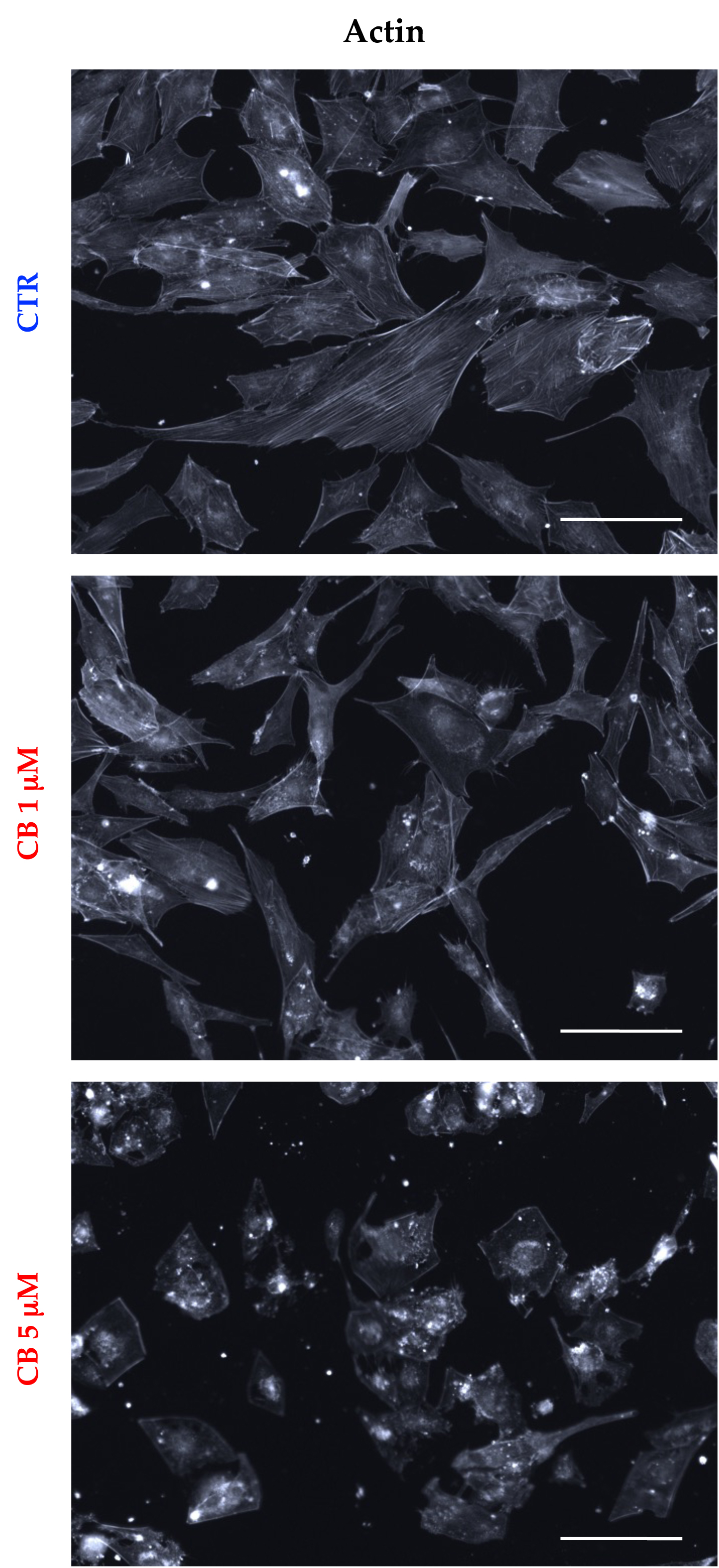

Supplement: Supplementary file 1 [file cells-11-01629-s001.zip › Supplementary Figure S4.jpg]

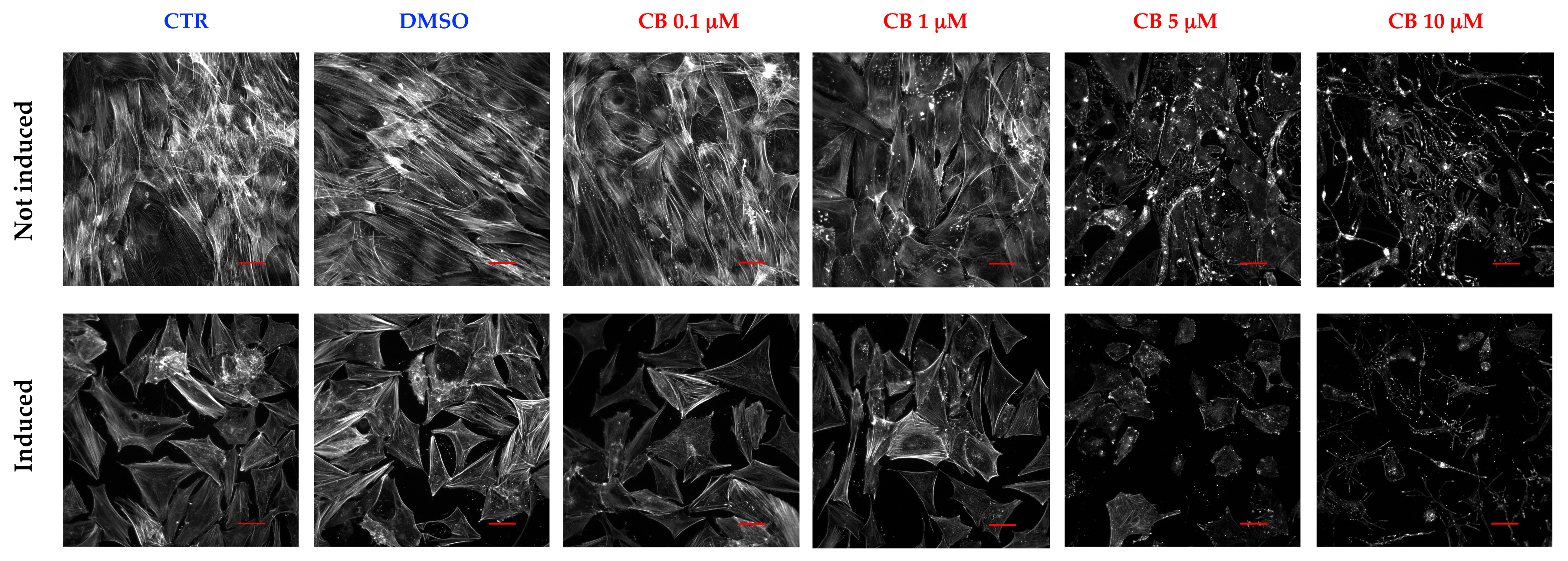

Supplement: Supplementary file 1 [file cells-11-01629-s001.zip › Supplementary Figure S5.jpg]
